# HP1021 is a redox switch protein identified in *Helicobacter pylori*

**DOI:** 10.1093/nar/gkab440

**Published:** 2021-06-17

**Authors:** Piotr Szczepanowski, Mateusz Noszka, Dorota Żyła-Uklejewicz, Fabian Pikuła, Malgorzata Nowaczyk-Cieszewska, Artur Krężel, Kerstin Stingl, Anna Zawilak-Pawlik

**Affiliations:** Department of Microbiology, Hirszfeld Institute of Immunology and Experimental Therapy, Polish Academy of Sciences, Wrocław 53-114, Poland; Department of Microbiology, Hirszfeld Institute of Immunology and Experimental Therapy, Polish Academy of Sciences, Wrocław 53-114, Poland; Department of Microbiology, Hirszfeld Institute of Immunology and Experimental Therapy, Polish Academy of Sciences, Wrocław 53-114, Poland; Department of Microbiology, Hirszfeld Institute of Immunology and Experimental Therapy, Polish Academy of Sciences, Wrocław 53-114, Poland; Department of Microbiology, Hirszfeld Institute of Immunology and Experimental Therapy, Polish Academy of Sciences, Wrocław 53-114, Poland; Department of Chemical Biology, Faculty of Biotechnology, University of Wrocław, Wrocław 50-383, Poland; Department of Biological Safety, National Reference Laboratory for Campylobacter, German Federal Institute for Risk Assessment, Berlin 12277, Germany; Department of Microbiology, Hirszfeld Institute of Immunology and Experimental Therapy, Polish Academy of Sciences, Wrocław 53-114, Poland

## Abstract

*Helicobacter pylori* is a gram-negative, microaerophilic, pathogenic bacterium and a widespread colonizer of humans. *H. pylori* has developed mechanisms that enable it to overcome the harsh environment of the human stomach, including reactive oxygen species (ROS). Interestingly, up to now no typical regulator dedicated to the oxidative-stress response has been discovered. In this work, we reveal that the inhibitor of replication initiation HP1021 functions as a redox switch protein in *H. pylori* and plays an important role in response to oxidative stress of the gastric pathogen. Each of the two predicted HP1021 domains contains three cysteine residues. We show that the cysteine residues of HP1021 are sensitive to oxidation both *in vitro* and *in vivo*, and we demonstrate that HP1021 DNA-binding activity to *oriC* depends on the redox state of the protein. Moreover, Zn^2+^ modulates HP1021 affinity towards *oriC* template DNA. Transcription analysis of selected *H. pylori* genes by RT-qPCR indicated that HP1021 is directly involved in the oxygen-dependent control of *H. pylori fecA3* and *gluP* genes, which are implicated in response to oxidative stress. In conclusion, HP1021 is a redox switch protein and could be a target for *H. pylori* control strategies.

## Graphical Abstract

**Graphical Abstract:**
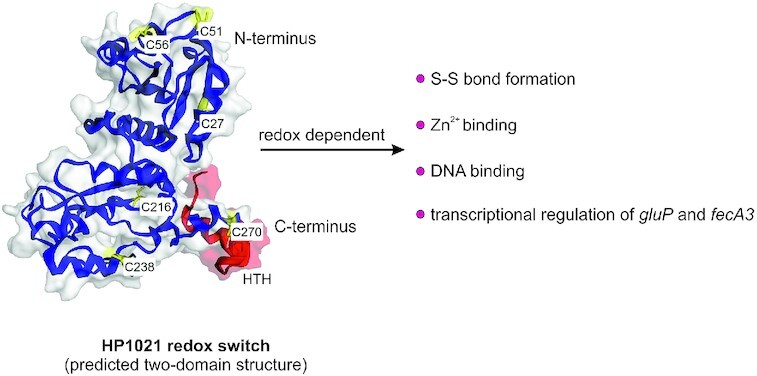
HP1021 functions as a redox switch protein in *H. pylori* and plays an important role in response to oxidative stress of the gastric pathogen.

## INTRODUCTION

*Helicobacter pylori* is a gram-negative, microaerophilic, pathogenic bacterium and a widespread colonizer of humans. Untreated, *H. pylori* infection usually lasts for life and increases the risk of peptic ulcer and gastric carcinoma development (1). *H. pylori* is one of the very few bacterial species that can survive in the harsh environment of the human stomach. In this environment, bacteria are exposed to detrimental factors, such as variable levels of acidity, occasionally reaching pH 2.5 and reactive oxygen species (ROS) produced as by-products of aerobic oxidation and metabolism or directly by host immune cells (1–3). Thus, *H. pylori* has developed mechanisms that enable it to respond to environmental stimuli effectively. However, the *H. pylori* repertoire of regulatory proteins is relatively small compared to that of other bacteria. According to the MiST database (4), the *H. pylori* genome encodes only approximately thirty signal transduction proteins, including two pleiotropic regulators, NikR and Fur, three two-component systems (FlgRS, ArsRS and CrdRS), two orphan regulators (HP1043 and HP1021) and a chemotactic system, Che (5–7). NikR and Fur control the transcription of genes critical for nickel and iron homeostasis, respectively, and genes involved in the bacterial response to acid stress, virulence factors, non-coding RNAs and toxin–antitoxin systems (6,8–11). FlgRS controls the transcription of flagellar genes (12–14); ArsRS regulates the transcription of genes involved in acid resistance (15–18); and CrdRS controls the transcription of genes important for copper homeostasis and numerous genes involved in a variety of cellular responses, including motility, ion transport and nitrosative stress responses (6,19,20). The orphan response regulator HP1043 (also known as homeostatic regulator HsrA) is an essential *H. pylori* gene. It has been shown to regulate the transcription of as many as 70 genes encoding proteins with disparate functions, such as protein synthesis, gene transcription, energy metabolism, chemotaxis and virulence (21–25). Therefore, HP1043 has been proposed to be a key regulator in *H. pylori*, likely involved in sensing and coordinating the response to environmental conditions. The HP1021 response regulator is not essential for *H. pylori* viability; however, the depletion of HP1021 reduces the *H. pylori* growth rate (26–28). HP1021 has been shown to control the transcription of 79 genes involved in many cellular activities, including metabolic processes, transcription and the synthesis of cofactors (27). HP1021 was also proposed to control the replication of the *H. pylori* chromosome by competing with the initiator DnaA protein for binding to the origin of chromosome replication (*oriC*). In turn, the initial DNA unwinding by DnaA is inhibited (28). Comprehensive biochemical studies have suggested that HP1021 activity is phosphorylation-independent (29). However, the exact mechanism of HP1021 regulation is unknown.

Interestingly, no typical regulator dedicated to the oxidative stress response has been discovered thus far in *H. pylori*. Oxidative stress is triggered by ROS, which induce damage to proteins, lipids, enzyme cofactors (Fe–S clusters) and DNA (via the Fenton reaction) (30). Therefore, bacteria encode several enzymes that help to neutralize ROS. However, these antioxidant systems must be adjusted to the ROS level by redox switches (e.g. OxyR and PerR) that transmit information on the redox state of the cell to mediate cellular processes and trigger appropriate cell responses (31,32). Redox sensors usually contain reactive, often reversibly oxidized, cysteine residues that change the conformation of the sensor protein, leading to altered activity (e.g. DNA binding, protein–protein interactions and chaperone activity). Additionally, redox sensor activity might be affected by the complexation of metal ions. For example, *Streptomyces coelicolor* RsrA and *Bacillus subtilis* SpxA bind Zn^2+^, *Escherichia coli* NorR and SoxR bind Fe^2+^ and Fe^2+^/Fe^3+^, respectively, *Pseudomonas syringae* CopC binds Cu^+^/Cu^2+^, and *B. subtilis* PerR binds Zn^2+^, Fe^2+^ or Mn^2+^ (32–35).

The *H. pylori* response to oxidative stress has also been extensively studied ((3,36,37) and references therein). *H. pylori* encodes proteins that directly detoxify ROS or regenerate ROS-modified proteins (KatA, SodB, AhpC, Tpx, BCP, MdaB and Trx), protect molecules from damage (Dps homolog NapA) or repair damaged molecules (e.g. MsrAB and MutY) (for details about *H. pylori* response to oxidative and nitrosative stress see a recent review (3)). Interestingly, in addition to the classical antioxidant defense mechanism, *H. pylori* uses chemotaxis to avoid ROS-abundant gastric microniches (38) or decreases the generation of NO by macrophages to attenuate the host immune response (39). It was also proposed that the pathogen incorporates large amounts of DNA by active uptake of free foreign DNA and uses it as a ‘shield’ to protect its own DNA from oxidative damage (40). However, *H. pylori* lacks common regulators for a response to oxidative stress, such as the redox switches OxyR and SoxR, the general stress response regulator RpoS and a glutathione–glutaredoxin reduction system (GSH/Grx).

In this work, we propose that HP1021 is a redox switch protein involved in the *H. pylori* response to oxidative stress. A structure prediction analysis suggested that HP1021 is composed of two functional domains, the N-terminal regulatory domain and the C-terminal domain containing a helix-turn-helix (HTH) DNA-binding motif. We show here that the cysteine residues of HP1021 are sensitive to oxidation both *in vivo* and *in vitro*. Consequently, HP1021 DNA-binding activity depends on the redox state of the regulator. Moreover, HP1021 activity is modulated by Zn^2+^. Transcription analysis of selected *H. pylori* genes indicated that HP1021 is involved in the control of the *H. pylori* response to oxidative stress.

## MATERIALS AND METHODS

### Materials and culture conditions

The strains, plasmids and proteins used in this work are listed in Supplementary Table S1. The primers used in this study are listed in Supplementary Table S2. *H. pylori* was cultivated at 37°C under microaerobic conditions (5% O_2_, 10% CO_2_ and 85% N_2_) generated by the jar evacuation-replacement method. *H. pylori* plate cultures were grown on Columbia blood agar base medium supplemented with 10% defibrinated horse blood (CBA-B) or 10% FCS (CBA-F). The liquid cultures were prepared in Brucella broth containing 10% fetal calf serum (BB-F) or 2% β-cyclodextrin (BB-C). All *H. pylori* cultures were supplemented with an antibiotic mix (41). The growth of the liquid cultures was monitored by measuring the optical density at 600 nm (OD_600_). *E. coli* was grown at 37°C on solid or liquid lysogeny broth supplemented with 50 μg/ml kanamycin or 100 μg/ml ampicillin where necessary. *E. coli* DH5α and BL21 were used for cloning and recombinant protein synthesis, respectively, while *E. coli* MC1061 was used for the propagation of plasmids used to transform *H. pylori*.

### Protein expression and purification

The purification of wild-type HP1021 and cysteine-less HP1021ΔCys variants of the recombinant Strep-tagged HP1021 proteins was carried out according to Strep-Tactin manufacturer's protocol (IBA Lifesciences). Briefly, BL21 cells (1 l) harbouring the expression vector (Supplementary Table S1) were grown at 37°C until an optical density (OD_600_) of 0.8 was reached. Then, protein synthesis was induced with 0.05 mM IPTG. The synthesis was continued for 3 h at 37°C and the cultures were harvested by centrifugation (10 min, 5 000 × *g* and 4°C). The cells were suspended in 20 ml of ice-cold sonication buffer W (100 mM Tris–HCl, pH 8.0; 300 mM NaCl and 1 mM EDTA) supplemented with complete, EDTA-free protease inhibitor Cocktail (Roche)), disrupted by sonication and centrifuged (30 min, 31 000 × *g* and 4°C). The supernatant was applied onto a Strep-Tactin Superflow high capacity Sepharose column (1 ml bed volume, IBA). The column was washed with buffer W until Bradford tests yielded a negative result (approximately 6 ml) and then washed with 5 ml of buffer W without EDTA. The elution was carried out with approx. 6 × 0.8 ml of buffer E (100 mM Tris–HCl, pH 8.0; 300 mM NaCl and 5 mM desthiobiotin). The fractions were stored at −20°C in buffer E diluted with glycerol to a final concentration of 50%.

### Zincon assay

To estimate the Zn^2+^-binding properties of HP1021, a competition assay with 2-carboxy-2′-hydroxy-5′-sulfoformazyl-benzene monosodium salt (Zincon) was performed. Stock solutions of Zincon (96440, Sigma Aldrich) were prepared in DMSO (9.25 mg/ml). The affinity of Zincon for Zn^2+^ was analyzed by titrating 100 μM Zincon in buffer Z (100 mM Tris–HCl, pH 8.0; 300 mM NaCl; 5 mM desthiobiotin and 10 μM ZnSO_4_, with 5 mM TCEP present when indicated) with HP1021 diluted in buffer E (see the protein expression and purification protocol), with 5 mM TCEP present when indicated, in a 1-cm path length quartz cuvette and reading the absorbance at 618 nm at 25°C using a Hitachi U-2900 spectrometer. The absorption of the Zn^2+^–Zincon complex was transformed to the species concentration using its molar absorption coefficient, 25 970 M^−1^ cm^−1^. The dissociation constant of the Zn^2+^–Zincon complex at pH 8.0 is 2.14 × 10^−7^ M, as previously determined (42).

### Isothermal titration calorimetry (ITC)

Binding of Zn^2+^, Co^2+^ and Ni^2+^ to HP1021 were monitored using the Nano-ITC calorimeter (TA Waters, USA) at 25°C with a cell volume of 1 ml. All experiments were performed in buffer E with 1 (Zn^2+^, Ni^2+^) or 3 mM (Co^2+^) TCEP. The HP1021 (titrate) concentration was 0.05 mM, whereas the metal (titrant) concentration was 0.5 mM. Concentrations of titrate and titrant were adjusted to obtain the best isotherms for proper analysis of equilibria and to highlight different processes throughout the titration experiment. After temperature equilibration, successive injections of the titrant were made into the reaction cell with 5.22 μl increments at 300 (Zn^2+^ and Ni^2+^) or 400 s (Co^2+^) intervals with stirring at 250 rpm. Control experiments to determine the heats of titrant dilution were performed using identical injections of Zn^2+^/Co^2+^/Ni^2+^ in the absence of protein. The net reaction heat was obtained by subtracting the heat of dilution from the corresponding total heat of the reaction. The titration data were analyzed using NanoAnalyze (version 3.3.0), NITPIC (version 1.2.7) (43) and SEDPHAT (version 15.2b) (44). Firstly, data were preprocessed using NanoAnalyze software dedicated to Nano-ITC calorimeter. Secondly, data integration and baseline subtraction were conducted using NITPIC freeware. Afterwards, integrated data were fitted with SEDPHAT.

### Surface plasmon resonance (SPR)

The Biacore system (Biacore T200, GE Healthcare) was used to study DNA-protein and protein–protein interactions by SPR (45). SPR response values are expressed in resonance units (RU), and for most molecules, 1 RU corresponds to the binding of 1 pg of the molecule per mm^2^ of the surface chip. The analysis was conducted following the protocols previously developed for determining DnaA-DNA interactions (46), with minor modifications. Biotinylated DNA fragments were obtained by polymerase chain reaction (PCR) with the following primer pairs: *oriC2* (P53-P54 using the pori2 plasmid template; 300 bp) and non-box DNA (P55-P56 using *H. pylori* 26659 genomic DNA template; 191 bp) (Supplementary Table S2). DNA was immobilized on a Series S Sensor Chip SA (approx. 90–140 RU) according to the supplier's instructions (GE Healthcare). The measurements were performed in Tris buffer (50 mM Tris–HCl, pH 8.0; 100 mM NaCl; and 0.2% Tween 20) at a continuous flow rate (15 μl/min). When indicated, *H. pylori* HP1021 and HP1021ΔCys protein samples were reduced with 5 mM TCEP or supplemented with ZnSO_4_. At the end of each cycle (180 s of the association phase followed by 180 s of dissociation), the bound proteins were removed by washing with 0.05% SDS (30 s), and the flow channels were equilibrated with Tris buffer until the baseline was stabilized. The BIAevaluation 4.1 and Excel 2019 software programs were used for the data analysis.

### Electrophoretic mobility shift analysis (EMSA)

The *oriC2* region was amplified by PCR using P17–P18 or P83–P18 primer pairs and a specific pori2 template (Supplementary Tables S1 and S2) giving FAM-*oriC2* or Cy5-*oriC2* probes. DNA probes representing putative promoter regions of selected genes and the control non-specific DNA fragment were amplified by PCR in two steps. In the first step, unlabeled DNA fragments were amplified using primer pairs: P67–P68 (26695 p*hyuA*), P69–P70 (N6 p*katA*), P73–P74 (N6 p*gluP*) and P75–P56 (26695 HP0180). Primers P68, P70, P74 and P75 contained overhangs complementary to P17 FAM–labeled primer. The unlabeled fragments were purified and used as templates in the second round of PCR using the FAM-labeled P17 primer and the unlabeled primer of each primer pair used in the first step. FAM-labeled DNA (5 nM) was incubated with the HP1021 protein at 37°C for 20 min in Tris buffer (50 mM Tris–HCl, pH 8.0; 100 mM NaCl and 0.2% Triton X-100). TCEP, metal ions (ZnSO_4_, NiCl_2_, CuCl_2_, Co(NO_3_)_2_), EDTA or DNA competitor (Poly(dA-dC)·Poly(dG-dT), P0307, Merck; 1:7.5 labeled DNA:competitor ratio of weight) were added to the reaction mixture when indicated. The complexes were separated by electrophoresis on a 4% polyacrylamide gel in 0.5 × TBE (1 × TBE: 89 mM Tris, 89 mM borate and 2 mM EDTA) or 0.5 × TB buffer (1× TB: 89 mM Tris, 89 mM borate) at 10 V/cm at room temperature (20–25°C). The gels were analyzed by a Typhoon 9500 FLA imager and ImageQuant software. The densitometric analysis of the gels was done using ImageLab (BioRad).

### Monitoring HP1021 thiol redox state *in vitro* and *in vivo*

To monitor HP1021 redox state *in vitro* and *in vivo*, a thiol-reactive probe 4-acetamido-4'-maleimidylstilbene-2,2'-disulfonic acid (AMS, A485, Thermo Fisher Scientific) was applied (47). *In vitro*, HP1021 was diluted in Tris buffer (2.72 μM, see EMSA for Tris buffer composition). When indicated, samples contained 2 mM tetramethylazodicarboxamid (TMAD, diamide), 5 mM TCEP or 6.25 μM metal ions (ZnSO_4_, NiCl_2_, CuCl_2_, Co(NO_3_)_2_). HP1021 oxidation by diamide was carried for 30 min on ice, while other samples were incubated for 30 min at 37°C. When indicated, protein samples (5 μl) were subsequently added to 10 μl of AMS solution (20 mM AMS; 50 mM Tris–HCl pH 7.5; 0,1% SDS and 10 mM EDTA) and incubated in the dark in a thermoblock for 30 min at 37°C. The samples were supplemented with Laemmli buffer without any reducing agent, heated at 95°C and separated by electrophoresis on a 10% SDS-PAGE gel. PageRuler Prestained Protein Ladder (Thermo Fisher Scientific) was used as a protein standard. The gels were transferred to nitrocellulose membranes and then incubated with an anti-HP1021 antibody (28) and anti-rabbit IgG (rabbit IgG HRP-linked whole Ab, NA934, GE Healthcare). The membranes were visualized upon chemiluminescent reaction, and the images were captured on X-ray film. *In vivo* AMS labeling was carried out according to the *E. coli* protocol (47), with the following modifications. *H. pylori* strains were cultured overnight in BB-C to OD_600_ ∼1.0 under microaerobic conditions. At time 0, the cultures were moved to the aerobic conditions and incubated at 37°C with shaking (N6 strain for 10 min, 26695 strain for 60 min). After the aerobic shock, the cultures were moved back to microaerobic conditions for 230 min (N6 strain) or 180 min (26695 strain). During the experiment, aliquots (1.8 ml) of cultures were taken at time points 0, 5, 10, 60 and 240 min. In addition, an aliquot of the culture grown under microaerobic conditions, supplemented with 10 mM DTT for 5 min, was taken and served as a control of a redox state of HP1021 in cells under fully reducing conditions. Aliquots of cultures were immediately mixed with 200 μl of 100% ice-cold trichloroacetic acid (TCA) to quench protein thiols and prevent cysteine shuffling (48), and incubated for 30 min on ice. All steps starting from TCA precipitation were done under aerobic conditions. After the 30 min incubation time, the samples were centrifuged (5 min, 15 000 rpm and 4°C), and the protein pellets were washed with 200 μl of cold 100% acetone, centrifuged (5 min, 15 000 rpm and 4°C) and dried at 37°C for 15 min. The pellets were suspended in AMS solution (20 mM AMS; 50 mM Tris–HCl pH 7.5; 0,1% SDS, and 10 mM EDTA) and incubated in the dark in a thermoblock mixer for at least 60 min (37°C, 1 400 rpm). The samples were further processed as described in the *in vitro* AMS protocol.

### Analysis of *H. pylori* gene transcription by RT-qPCR

*H. pylori* was grown under microaerobic conditions (12 ml) to the logarithmic phase (OD_600_ ∼1.0), and then, the cultures were moved to aerobic conditions (normal atmosphere at 37°C with orbital shaking) for 20 min. Samples (1 ml) were collected by centrifugation (13 000 × *g*, 15 s, room temperature) before and after oxidative stress induction. Then, the cell pellet was suspended in Fenozol Plus (A&A Biotechnology). RNA was extracted using a Total RNA Mini Plus kit (A&A Biotechnology) according to the manufacturer's protocol and further treated with RNase-free DNase I (Thermo Scientific). The RNA quality and quantity were determined by a NanoDrop Lite spectrophotometer and agarose gel electrophoresis. Reverse transcription (RT) reactions were carried out using 0.5 μg of total RNA in a 20 μl volume reaction mixture of iScript cDNA Synthesis Kit (Bio-Rad), as described by the manufacturer. The mRNA levels of the selected *H. pylori* genes were quantified by qPCR, which was performed on a CFX96 Touch Real-Time PCR detection system (Bio-Rad) using SensiFAST SYBR No-ROX (Bioline) and the following parameters: 95°C for 3 min, followed by 40 three-step amplification cycles consisting of 5 s at 95°C, 10 s at 60°C and 20 s at 72°C. The reaction mixtures (15 μl) contained qPCR mix (7.5 μl), cDNA (2 μl of the RT reaction mixture diluted 10×) and primers (0.4 μM each). The following primer pairs were used: P25–P26, *gluP* (HP1174 in *H. pylori* 26695); P27–P28, *fecA3* (HP1400 in *H. pylori* 26695); P21–P22, *katA* (HP0875 in *H. pylori* 26695); and P35–P36, 16S rRNA. The relative quantity of mRNA for each gene was determined by referring to the mRNA levels of *H. pylori* 16S rRNA.

## RESULTS

### HP1021 DNA binding activity depends on redox conditions

**Figure 1.**
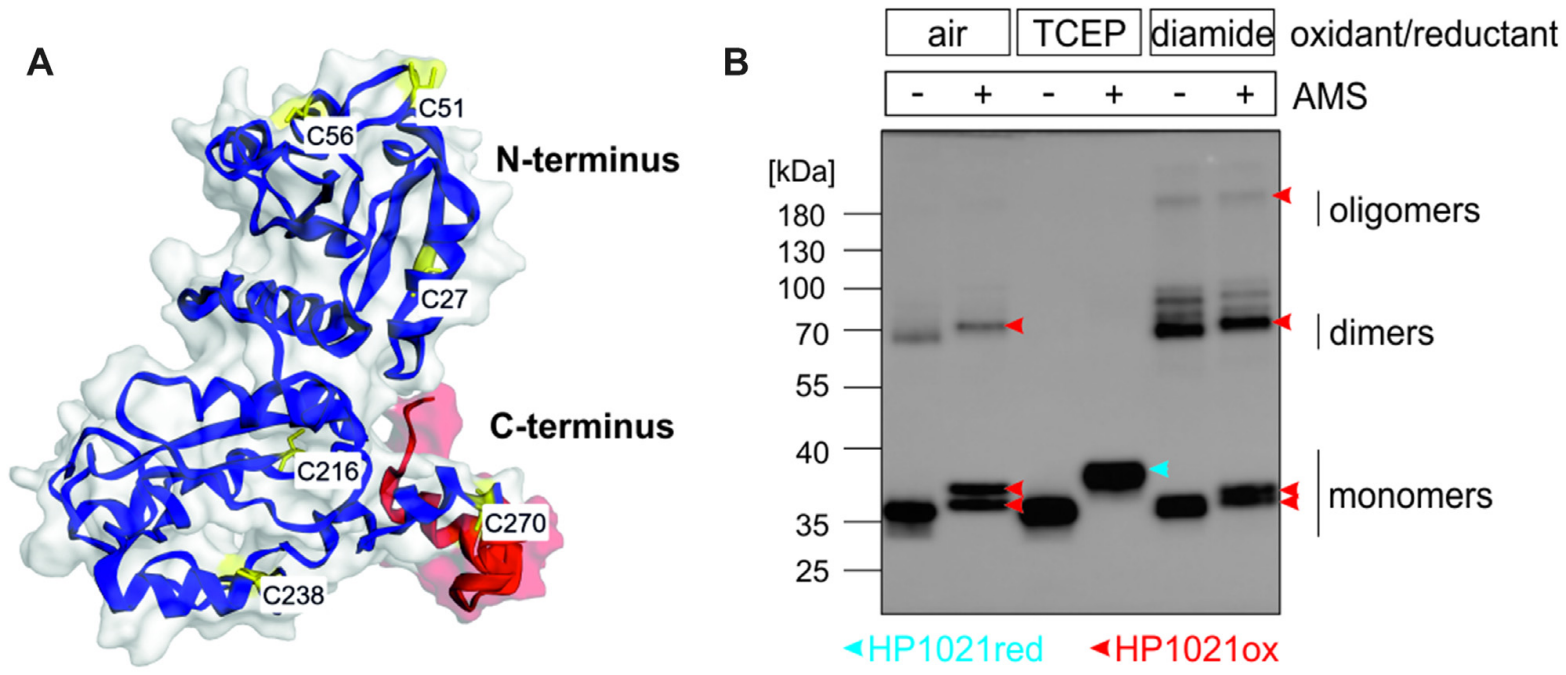
The *H. pylori* HP1021 protein. (**A**) A model of the HP1021 protein structure predicted by Phyre^2^. Cysteine residues are highlighted in yellow, while the HTH motif is red. (**B**) Analysis of the thiol redox state of HP1021 incubated with reducing or oxidizing agents. HP1021 (2.72 μM) was incubated in Tris buffer containing TCEP or diamide or without any reducing or oxidizing agents. Where indicated, samples were subsequently labeled with AMS. HP1021 was resolved in a 10% SDS-PAGE gel under non-reducing conditions, transferred onto PVDF membrane and detected by a rabbit polyclonal anti-6HisHP1021 IgG. Digital processing was applied equally across the entire image, including controls.

A structure prediction analysis, performed with Phyre2 ((49), see Supplementary Information for details), suggested that HP1021 (298 amino acids and 35.2 kDa) is composed of two functional domains, the N-terminal regulatory domain and the C-terminal domain containing a helix-turn-helix (HTH) DNA-binding motif (Figure 1A). Each of the two domains contains three cysteine residues (C27, C51, C56 and C216, C238, C270, respectively). BLAST analysis of the HP1021 amino acid sequence from 486 *H. pylori* strains indicated that the number and position of the cysteine residues are conserved (Supplementary Figure S1).

Using a cysteine-reactive probe AMS (Materials and Methods) we analyzed whether cysteine residues in a recombinant, purified HP1021 protein are redox-active. AMS exclusively reacts with reduced cysteine residues and increases the molecular mass of a labelled protein by 0.5 kDa for each fully reduced cysteine. We labeled three protein fractions: (i) TCEP-reduced (HP1021red), (ii) air-oxidized (HP1021ox) and (iii) diamide-oxidized (HP1021dia). The AMS-treaded HP1201 protein from each fraction migrated slower than untreated HP1021; thus, the protein was successfully labelled by AMS. All HP1021red and the majority of the HP1021ox protein migrated as monomers (Mw ∼35–40 kDa) (Figure 1B), while some of the HP1021ox and HP1021dia protein formed higher molecular weight complexes (dimers and oligomers). Each of the monomeric, AMS-labeled HP1021ox or HP1021dia had lower molecular weight than the AMS-labelled HP1021red, which indicated that fewer AMS molecules reacted with oxidized HP1021 than with the reduced HP1021 protein. Thus, we assumed that HP1021red represented a fully reduced protein while in HP1021ox or HP1021dia some cysteine residues were oxidized. Thus, oxidation *in vitro* leads to cysteine residue oxidation resulting in inter- and possibly intra-molecular disulfide bridge formation. Diamide, a strong and specific cysteine-residue oxidizer, increased the number of oxidized cysteine residues compared to the air-oxidized protein fraction. Thus, the oxidizing agents may differently shape the molecular structure of the oxidized HP1021. Further studies are required to determine which oxidation pattern(s) may happen *in vivo* (see also below). Nonetheless, we conclude that HP1021 contains redox-reactive cysteine residues, which may control HP1021 activity in response to oxidative stress.

**Figure 2.**
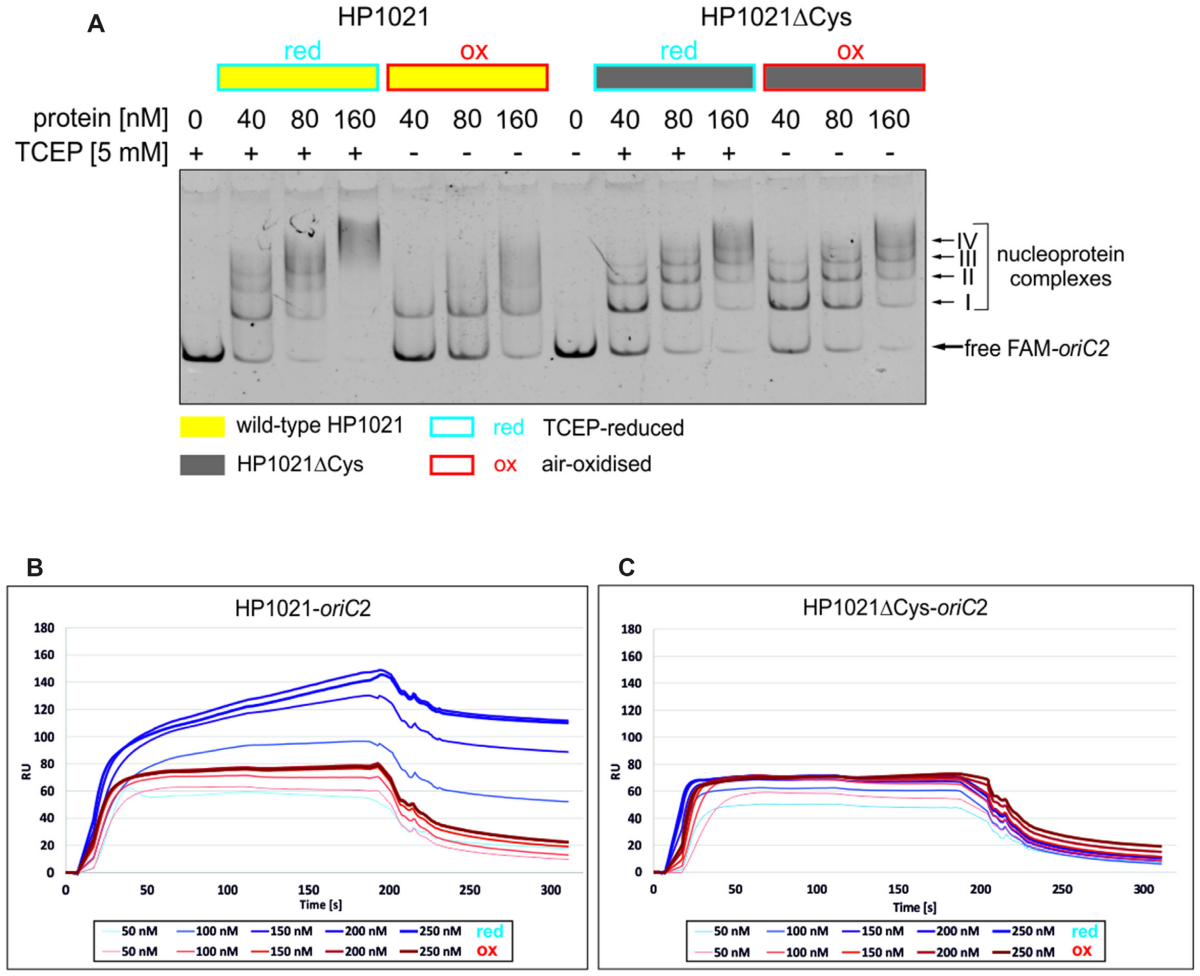
Influence of the redox state of HP1021 on its DNA-binding activity. (**A**) EMSA was performed using the FAM-labeled *oriC2* fragment, which was incubated with the indicated amounts of HP1021 protein variants in the presence or absence of TCEP. Digital processing was applied equally across the entire image, including controls. (**B, C**) SPR analysis of the interactions between the HP1021 protein variants and *oriC2* in the presence or absence of TCEP. The protein concentrations are marked with lines of different thickness and different shades of red (HP1021ox) and blue (HP1021red). RU, response units.

HP1021 binds DNA and regulates the transcription of genes (27). The response regulator may also control the initiation of *H. pylori* chromosome replication by binding to the *oriC2* subregion containing three HP1021 boxes (28). Since HP1021 was shown to be sensitive to oxidation, we analyzed how the redox state affects HP1021 DNA-binding activity using an electrophoretic mobility shift assay (EMSA) and surface plasmon resonance (SPR) (Materials and Methods). For the EMSA, we analyzed the interactions of TCEP-reduced and air-oxidized HP1021 (HP1021red and HP1021ox, respectively) with the FAM-labeled *oriC*2 DNA probe (FAM-*oriC2*) (Figure 2A). HP1021red interacted with *oriC2* similarly, as shown by previously published reports (28). It formed distinct nucleoprotein complexes (types I-IV) at lower protein concentrations (40–80 nM), and undefined high-molecular-weight complexes at higher HP1021 concentrations (160 nM). HP1021ox exhibited a 2- to 4-fold lower affinity towards *oriC2* than did the HP1021red protein (40 nM HP1021red and 80–160 nM HP1021ox bound approximately the same amount of *oriC2*). In addition, only one predominant type of nucleoprotein complex was formed by HP1021ox (type I), which possibly represented the most stable or the least redox-sensitive complex (Figure 2A). These results indicated that HP1021 DNA-binding activity is affected by the redox state of the cysteine residues.

To determine whether cysteine residues are involved in the direct binding of the protein to DNA or are alternatively involved in the modulation of the protein structure, we produced and purified a recombinant, strep-tagged HP1021ΔCys protein—a version of HP1021 in which all cysteine residues were exchanged for alanine residues (Materials and Methods, Supplementary Figure S2). According to the EMSA results, HP1021ΔCys bound FAM-*oriC*2 with an affinity similar to that of the HP1021red protein irrespective of the presence or absence of TCEP (Figure 2A). Thus, cysteine residues are not necessary to establish direct contact with DNA. However, our results indicate that cysteine residues are specifically required to control the conformational changes of the protein, possibly via the formation of intramolecular cysteine bridge(s) (Figure 1), which affects protein DNA-binding activity.

To confirm our results, we used SPR, which allows measuring HP1021–*oriC2* interactions in real-time in a more quantitative manner than does EMSA (Figure 2BC). The SPR results indicated that HP1021red binds *oriC2* more efficiently (up to 150 RU at 200–250 nM) than does its oxidized version (up to 80 RU at 150–250 nM). Thus, approx. 50% more HP1021red molecules than HP1021ox bound to chip-immobilized *oriC2*. Moreover, HP1021red dissociated from *oriC2* slower than did HP1021ox, which indicates that the stability of the HP1021red-*oriC2* complex is greater than that of the HP1021ox-*oriC2* complex. Mutated HP1021ΔCys bound *oriC2* and dissociated from DNA, irrespective of the redox state. Therefore, the SPR analysis results agree with the EMSA results, indicating that the redox state of the HP1021 protein is important for its interactions with DNA.

### HP1021 binds divalent cations

**Figure 3.**
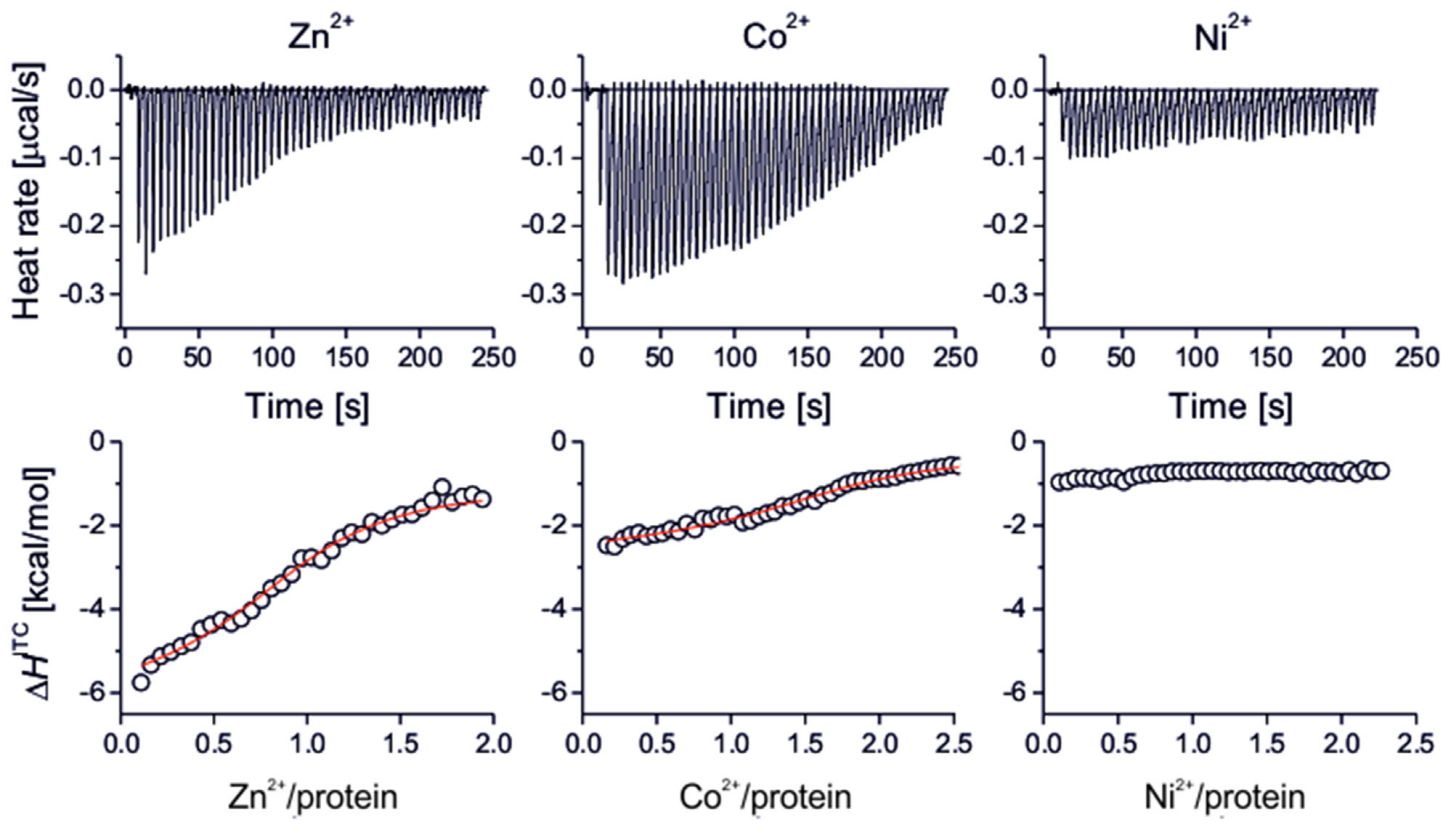
ITC analysis of Zn^2+^, Co^2+^ and Ni^2+^ binding to HP1021. The top panels show baseline-subtracted thermograms. The bottom panels represent the binding isotherms. All measurements were obtained in buffer E (100 mM Tris–HCl; pH 8.0; 300 mM NaCl and 5 mM desthiobiotin) at 25°C in the presence of TCEP (1 mM (Zn^2+^, Ni^2+^) or 3 mM (Co^2+^)).

Cysteine residues are not only redox-sensitive but also play important roles in the coordination of divalent metal ions (e.g. Zn^2+^, Cu^2+^, Ni^2+^, Co^2+^), which are important for the structure and/or catalytic activity of a given protein (50,51). We used isothermal titration calorimetric (ITC) assay to study the interactions between recombinant, purified HP1021 and metal ions. We tested only Zn^2+^, Co^2+^, Ni^2+^, which are located in the stronger binding end of the Irving–Williams series and are bound by the proteins with high affinities (51). We did not test highly redox reactive metal ions such as Cu^+^/Cu^2+^ and Fe^2+^/Fe^3+^ due to their impact on cysteine residues and required totally oxygen-free conditions (52). The redox reactivity of Co^2+^ and Ni^2+^ was controlled by the presence of TCEP during titration. Importantly, it was shown that divalent metal ions do not interact tightly with TCEP (53) with the exception of Co^2+^, which demonstrates a moderate affinity for TCEP and Tris-HCl buffer as observed in ITC experiments. The comparison of ITC thermograms of HP1021 titrated with Zn^2+^, Co^2+^ and Ni^2+^ (Figure 3) indicated that only Zn^2+^ and Co^2+^ interact with HP1021 protein, while no Ni^2+^ binding was observed (Figure 3, bottom panel - lower Δ*H*^ITC^ changes indicates weaker coordination of metal ion to protein molecules). Binding isotherms plotted as a metal-to-protein molar ratio show that Zn^2+^ interacts with stoichiometry 1:1, while for Co^2+^ this stoichiometry is slightly higher. Fitted dissociation constants (red lines) for those ions are almost identical and are 4.7 × 10^−6^ and 4.0 × 10^−6^ M, respectively. However, it should be underlined that unspecific interaction of Co^2+^ with TCEP and Tris–HCl may somehow affect obtained constant value. Co^2+^ is isostructural to Zn^2+^ metal ion and is commonly used for studying Zn^2+^-binding sites in proteins due to d-d transitions (in contrast to Zn^2+^) observable in UV-Vis spectra which inform on ligand composition and geometry of metal-binding site (54). It is also known that Co^2+^ forms weaker complexes to Zn^2+^ what is here somehow confirmed by a significantly lower change of Δ*H*^ITC^. This parameter does not change for Ni^2+^. To conclude, HP1021 binds Zn^2+^ specifically with the highest affinity, while Co^2+^ and Ni^2+^ are bound either with lower affinity or do not bind at all.

**Figure 4.**
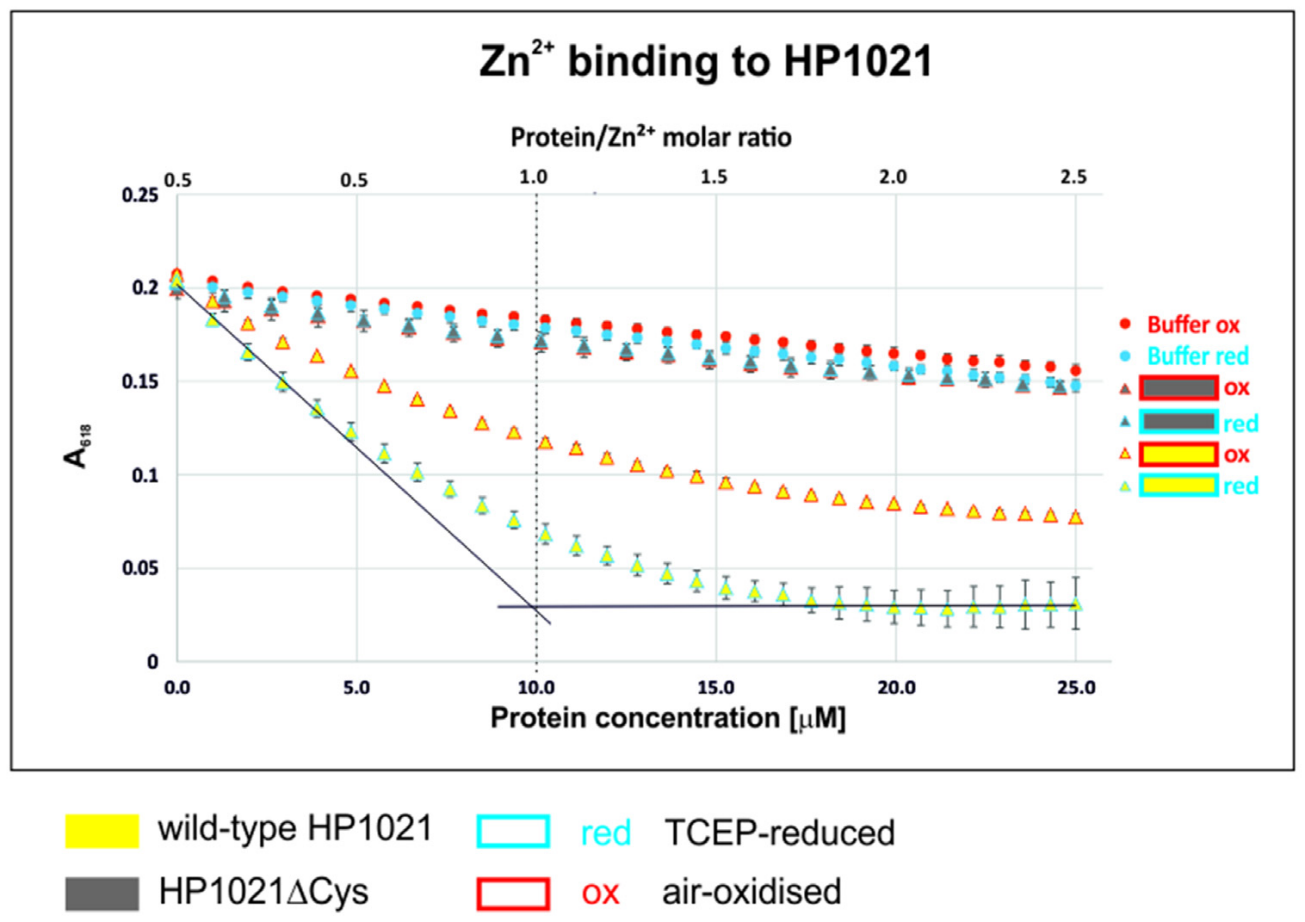
Zn^2+^ binding to HP1021 and HP1021ΔCys. Reduced and non-reduced proteins were gradually added to a Zn^2+^–Zincon complex. The binding of Zn^2+^ to the proteins was analyzed by complex characteristic absorption decreases at 618 nm. The buffer without protein was used as a control. A dotted line indicates the 1:1 protein: Zn^2+^ molar ratio.

Next, to further analyze Zn^2+^ binding by HP1021, we used a Zincon competition assay ((42), Materials and Methods). Zincon is a metallochromic indicator that, upon metal binding, shifts the optical absorption from 488 nm to ∼620 nm. It competes with a protein for metal; therefore, it is applied to multiple studies of Zn^2+^ binding and dissociation from proteins; (42) provides an example. We titrated partially saturated Zincon with Zn^2+^ (Zn^2+^–Zincon complex) by the HP1021 TCEP-reduced and air-oxidized proteins. We observed that absorption at 618 nm decreased systematically with increasing concentration of the HP1021red protein (Figure 4, yellow triangles, blue outline). This indicated that the reduced HP1021 protein bound Zn^2+^, which confirmed our ITC results. Under similar conditions, the air-oxidized HP1021 protein also competed with Zincon for Zn^2+^ but with much lower efficiency (Figure 4, yellow triangles, red outline). As expected, HP1021ΔCys did not efficiently compete with Zincon for Zn^2+^ (gray triangles, red and blue outlines). Altogether, the Zincon assay proved that HP1021 binds Zn^2+^ in a cysteine-dependent manner and that HP1021-Zn^2+^ interactions are related to the redox state of HP1021.

### Divalent cations modulate HP1021 DNA-binding activity

**Figure 5.**
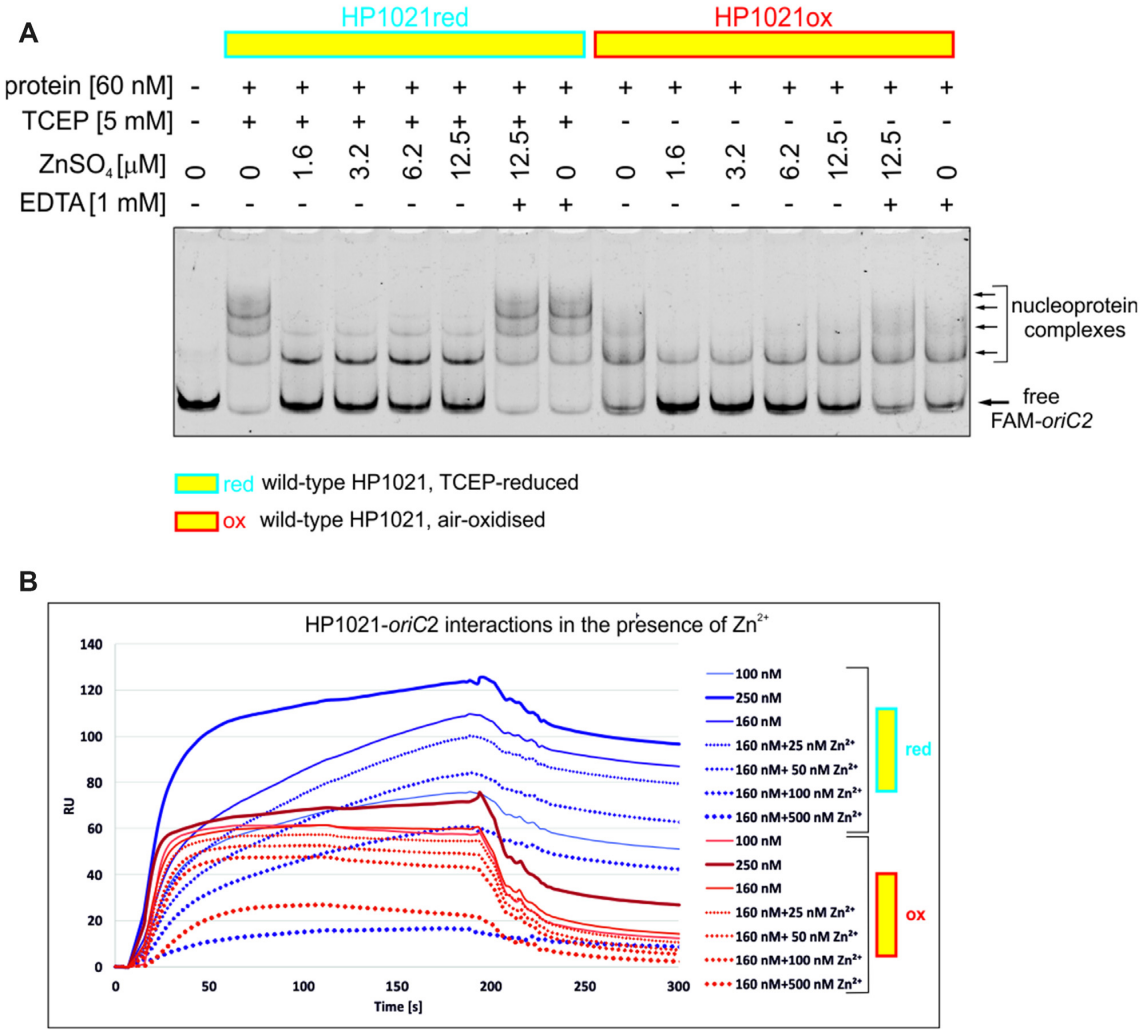
Influence of Zn^2+^ on HP1021 DNA-binding activity. (**A**) A gel-retardation assay was performed using the FAM-*oriC2* fragment that had been incubated with the indicated amounts of the HP1021 protein variants in the presence or absence of Zn^2+^. Digital processing was applied equally across the entire image, including controls. (**B**) SPR analysis of the interactions between HP1021 and *oriC2* in the presence or absence of Zn^2+^. RU, response units.

**Figure 6.**
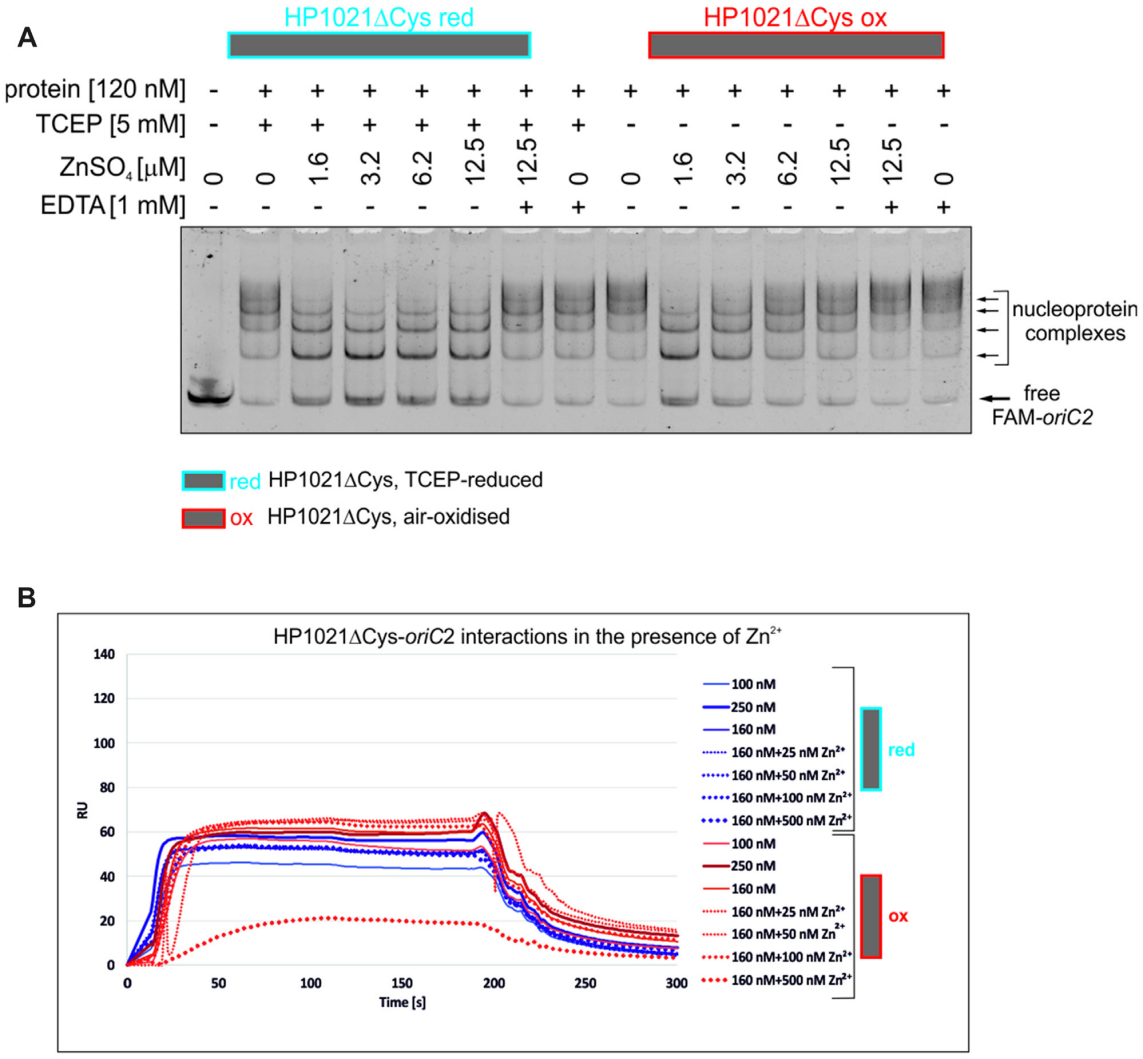
Influence of Zn^2+^ on HP1021ΔCys DNA-binding activity. (**A**) The gel-retardation assay was performed using the FAM-*oriC2* fragment that had been incubated with the indicated amounts of the HP1021ΔCys protein variants in the presence or absence of Zn^2+^. Digital processing was applied equally across the entire image, including controls. (**B**) SPR analysis of the interactions between HP1021ΔCys and *oriC2* in the presence or absence of Zn^2+^. RU, response units.

Next, we sought to determine whether Zn^2+^ binding affects HP1021-DNA interactions. We incubated HP1021red and HP1021ox with *oriC2* in the presence and absence of Zn^2+^ and analyzed the nucleoprotein complexes with the EMSA. We observed that Zn^2+^ reduced the affinity of HP1021red for *oriC2;* only the complex I was still stable in the presence of Zn^2+^ (Figure 5A). The partial stability of the complex I resulted from the presence of EDTA in the TBE running buffer because when EDTA, a metal chelating agent, was eliminated from the TBE running buffer, the stability of this complex was further diminished by Zn^2+^ (Supplementary Figure S3; please note that resolution of the gels run in TB buffer (without EDTA) was lower than that of gels run in TBE buffer; thus we present results of the TB gels as complementary to standard gels resolved in TBE buffer). Consequently, upon the chelation of Zn^2+^ by EDTA during DNA binding reaction (20 min at 37°C), the affinity of HP1021red for *oriC*2 was fully restored (Figure 5A). Zn^2+^ also destabilized HP1021ox – *oriC2* interactions. The weak effect of enhancing the interaction of HP1021ox (and HP1021ΔCys, see below) with *oriC2* along with the increase in ZnSO_4_ concentration was probably caused by the interaction of Zn^2+^ ions with amino acid residues other than cysteine residues (55) or interactions of SO_4_^2–^ ions with HP1021. As previously observed, the affinity of HP1021ox for DNA was lower; thus, the Zn^2+^ effect was more pronounced for HP1021red than for HP1021ox. The EMSA results were also verified by the SPR analysis. Consistently, the presence of Zn^2+^ reduced the affinity of HP1021red for *oriC*2 in a Zn^2+^ concentration-dependent manner (Figure 5B). We also observed that the addition of Zn^2+^ inhibited HP1021ox-*oriC*2 interactions, but as observed with the EMSA, this effect was less pronounced than for HP1021red-*oriC*2 interactions. Using HP1021ΔCys (Figure 6AB), we observed only a marginal reduction in HP1021ΔCys binding to *oriC2* in the presence of Zn^2+^, confirming the important role of cysteine residues in the efficient binding of Zn^2+^ (Figure 4) and the role of Zn^2+^ binding in HP1021 on HP1021 interactions with DNA (Figures 5 and 6).

ITC analysis indicated that metal ions other than Zn^2+^ may also interact with HP1021; therefore, we used EMSA to test if Ni^2+^, Co^2+^ and Cu^2+^ can also destabilize HP1021-*oriC2* complexes. The HP1021-*oriC2* complexes were formed in the presence or absence of metal ions and then resolved in polyacrylamide gels in 0.5 × TBE or 0.5 × TB buffers. Cu^2+^, which usually tightly binds to metalloproteins, had only a marginal influence on the complexes' stability (Supplementary Figure S4A, B). Ni^2+^ and Co^2+^ partially destabilized the complexes (Co^2+^ > Ni^2+^), but none of them fully destabilized the complexes as Zn^2+^ did (Supplementary Figure S4AB, 0.5 × TB buffer, 6.25 μM Zn^2+^, Ni^2+^ and Co^2+^). None of the metal ions oxidized HP1021 under test conditions (in the presence of 5 mM TCEP, Supplementary Figure S4C); thus, destabilization of HP1021-*oriC2* complexes was possibly driven by structural changes in HP1021 resulting from metal binding.

Altogether, our EMSA, ITC and Zincon results indicated that HP1021 binds divalent transition metal ions with Zn^2+^ being bound with the highest affinity. Accordingly, Zn^2+^ exhibits the highest potential to affect HP1021red-*oriC2* interactions.

### Redox states of native HP1021 in *H. pylori* cells

**Figure 7.**
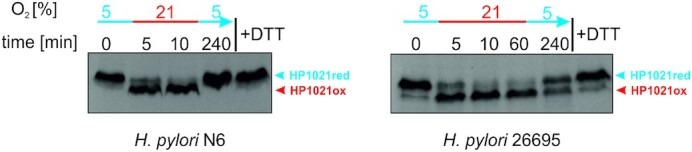
Redox states of HP1021 in the *H. pylori* N6 and 26695 wild-type strains. The same bacterial culture was subsequently incubated under different oxygen conditions, as indicated above the gels. Cells collected at the indicated time points were treated with 10% TCA, followed by alkylation with AMS. Cellular proteins, including the reduced, DTT-treated and AMS-labeled controls, were separated by 10% SDS-PAGE under non-reducing conditions and subjected to Western blot analysis using a rabbit polyclonal anti-6HisHP1021 IgG. Each lane contains proteins isolated from the same amount of bacteria (∼5 × 10^8^ cells). Digital processing was applied equally across the entire image, including controls.

To test the redox state of HP1021 directly in *H. pylori* cells and its putative change upon exposure to aerobic stress, we labelled *H. pylori* proteins by AMS (Materials and Methods and (47)). Briefly, *H. pylori* was grown under standard microaerobic conditions to the logarithmic phase of growth, then moved to aerobic conditions for the indicated period and transferred back to microaerobic conditions (see Materials and Methods for details). To establish a control for the completely reduced HP1021, we used *H. pylori* cells grown under microaerobic conditions and treated them with DTT before AMS labelling. At the indicated time points (Figure 7 and Supplementary Figure S5), samples of the *H. pylori* culture were precipitated with TCA, and total precipitated *H. pylori* proteins were labelled by AMS. *H. pylori* proteins were separated by non-reducing SDS-PAGE, and HP1021 was detected by anti-HP1021 antibodies as previously described (28). In the *H. pylori* N6 and 26695 cells grown under microaerobic conditions, the HP1021 cells and DTT-treated controls migrated similarly (Figure 7 and Supplementary Figure S5). Thus, under microaerobic conditions, HP1021 in the *H. pylori* cells was almost exclusively reduced. The HP1021 protein detected in the *H. pylori* cells moved to aerobic culture conditions migrated faster than that from the cells grown microaerobically. This finding indicated that HP1021 was not labelled or less efficiently labelled by AMS than was the HP1021 protein detected in the cells grown microaerobically. We conclude that HP1021 had become oxidized at cysteine residues when moved from the 5% to 21% O_2_ atmosphere. We did not observe any HP1021 dimers, indicating that no intermolecular cysteine bridges were formed (Supplementary Figure S5); thus, cysteine residue oxidation in HP1021 *in vivo* possibly leads to intramolecular bridge formation. The oxidation occurred within the initial 5 min of incubation at 21% O_2_, and the protein stayed in the oxidized form for the entire period it was exposed to air. In cells reverted to microaerobic culture conditions, the reduced state of the HP1021 protein was partially restored. Thus, we concluded that the cysteine residues of HP1021 are sensitive to oxidation in *H. pylori* cells. HP1021 becomes oxidized upon exposure to oxygen stress, while the reduced form is restored after the cells are re-exposed to microaerobic culture conditions.

### HP1021 controls the transcription of specific genes in an oxygen-dependent manner

Previous studies showed that the phenotype of the HP1021 deletion mutant depended on the parental *H. pylori* strain used for mutagenesis (26,27). Results of microarray analysis, that was performed by Pflock and co-workers to determine the *H. pylori* gene transcription in the 26695 wild-type (WT) strain and compare it with that of the isogenic *H. pylori* 26695/HP1021::km mutant, revealed that HP1021 activates the transcription of 51 genes while repressing the expression of 28 genes (27). However, the signalling that controls HP1021 activity was not identified. Our analyses indicate that HP1021 may respond to oxidative stress. Thus, to better characterize the HP1021 response to oxidative stress in the cultured *H. pylori* cells, using two parental strains, N6 and 26695, we constructed *H. pylori* strains lacking HP1021 (ΔHP1021) and HP1021-complemented strains (COM) (Supplementary Figure S11B, C). In addition, we constructed two other types of strains: (i) COM ΔCys strain in which ΔHP1021 strains were complemented by the mutated HP1021 gene encoding a cysteine-less protein variant, and (ii) ΔCys strain in which the mutated gene encoding a cysteine-less protein variant replaced HP1021 in the WT strain (Supplementary Materials and Methods, Supplementary Figures S2 and S11ED). We took care to minimize the number of passages between the mutagenesis steps to reduce the incidence of possible suppressor mutations. The expression of HP1021 in the mutated strains was analyzed by Western blotting. No HP1021 was observed in ΔHP1021 strains, while in COM, COM ΔCys and ΔCys strains the expression of HP1021 was comparable to that in the wild-type strain (Supplementary Figure S6). We compared the growth of the mutant strains with *H. pylori* wild-type strains in liquid culture under microaerobic conditions. The growth of the ΔHP1021 strains was retarded compared to the growth of the wild-type strains, as observed previously (26–28). Generation times (G) of the N6 ΔHP1021 and 26695 ΔHP1021 mutants were 269 ± 15 min and 305 ± 22 min, respectively, while the G of the WT strains were 171 ± 7 min (N6) and 152 ± 10 min (26695) (Supplementary Figure S7AB). The COM, COM ΔCys and ΔCys strains grew similarly to the cognate WT strains, which indicates that wild-type or cysteine-less mutant HP1021 complemented growth defects of ΔHP1021 strains.

**Figure 8.**
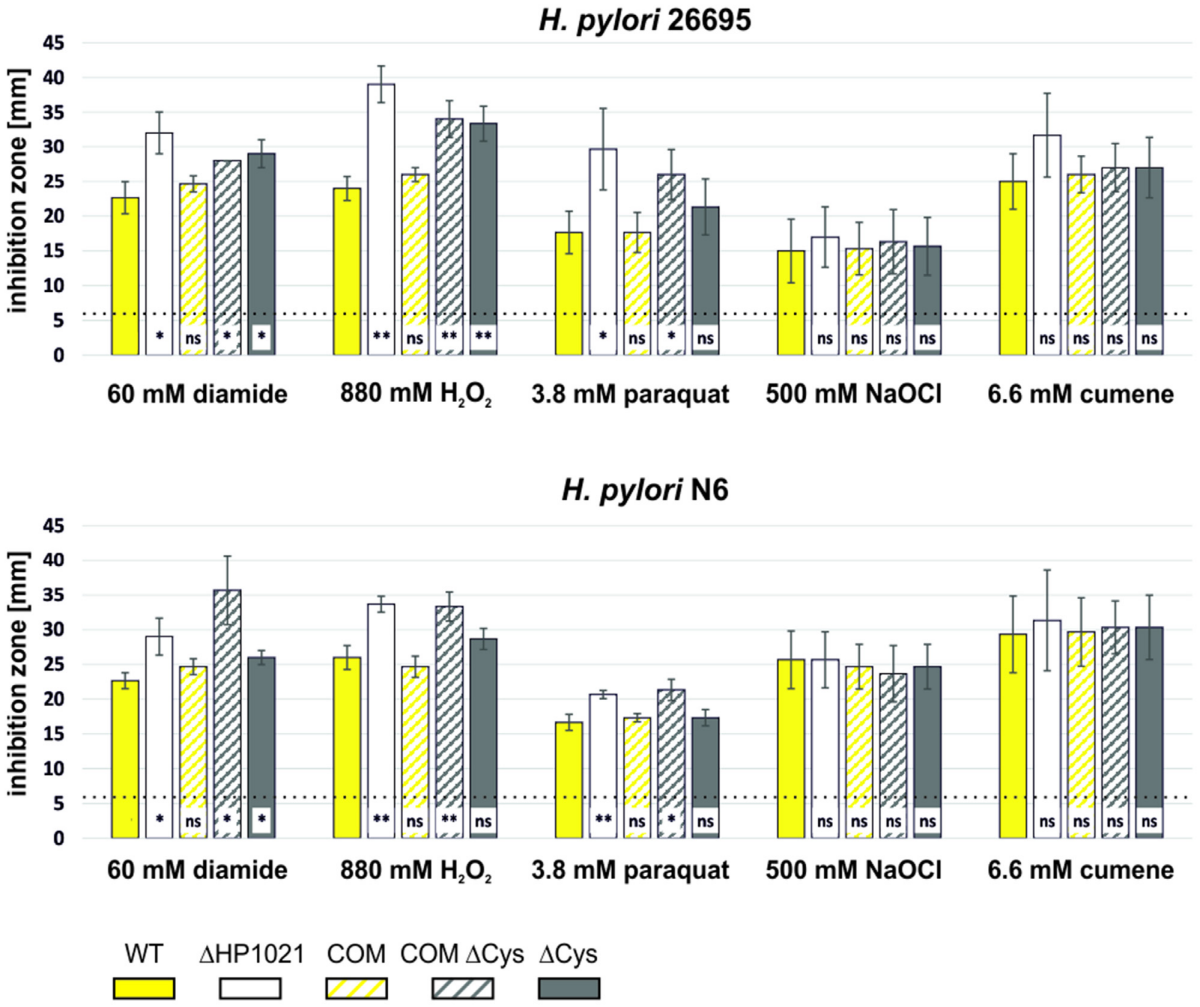
*H. pylori* susceptibility to oxidative agents analyzed by the disc diffusion assay. Bacteria were evenly spread on CBA-F agar plates. Glass fibre discs were soaked with oxidizing solutions and placed on the plates. The diameter of the inhibition zone around the discs was determined after 5 days of incubation under microaerobic conditions. Data present mean values and standard deviations of three independent assays. Significance values were calculated using a two-tailed Student's *t*-test. ns, not significant; *P < 0.05; **P < 0.01; ***P < 0.001; dotted line – diameter of the discs.

To test whether HP1021 helps *H. pylori* respond to oxidative stress, we applied a disc diffusion assay (Materials and Methods). We tested *H. pylori* growth in the presence of five different oxidizers: diamide (inducing general disulfide stress), H_2_O_2_ (inorganic peroxide), cumene hydroperoxide (organic hydroperoxide), paraquat dichloride (generating O_2_^•-^), and sodium hypochlorite (a source of hypochlorous acid). In each strain and oxidizer, the sensitivity of WT and COM strains were comparable; thus, HP1021 complemented the ΔHP1021 strain (Figure 8). Mutant ΔHP1021, COM ΔCys and ΔCys of both strains were more sensitive to diamide than were the cognate wild-type and COM strains. It indicated that HP1021, via cysteine-dependent response, is important to overcome the stress that disturbs the cellular thiol-disulfide redox balance and may trigger a global transcriptomic response (56,57). 26695 ΔHP1021, COM ΔCys and ΔCys strains and N6 ΔHP1021 and COM ΔCys were more sensitive to H_2_O_2_ than the WT and COM strains. When sensitivity to paraquat was analyzed, *H. pylori* ΔHP1021 and COM ΔCys were more sensitive than WT and COM strains. The sensitivity of HP1021 mutant strains to cumene hydroperoxide or sodium hypochlorite was similar to that of the WT and COM strains; therefore, HP1021 is possibly not involved in sensing hypochlorous acid and organic hydroperoxides (Figure 8). Thus, we conclude that HP1021 responds to reactive oxygen species: O_2_^•-^ and H_2_O_2_.

O_2_^•-^ (which undergoes dismutation to H_2_O_2_) and H_2_O_2_ are oxidants which, unless decomposed by a catalase, may eventually be transformed into highly toxic hydroxyl radical (30). Thus, catalase plays an important role in O_2_^•-^ and H_2_O_2_ detoxification. Pflock and co-workers had shown that both *katA* transcription and catalase synthesis were reduced in the 26695/HP1021::km strain in comparison to the wild-type strain (27). Thus, we tested catalase activity in HP1021 mutant strains. Catalase activity was detected in N6 and 26695 WT strains (Supplementary Figure S8). In ΔHP1021 strains catalase activity was reduced (N6ΔHP1021) or not detectable (26695ΔHP1021), while it was restored in COM strains. In COM ΔCys and ΔCys strains catalase activity was observed, albeit slightly reduced compared to the wild-type strains. Our results corresponded with the results of the disc diffusion assay, further supporting our hypothesis that HP1021 is important for *H. pylori* response to reactive oxygen species, in particular O_2_^•-^ and H_2_O_2_.

**Figure 9.**
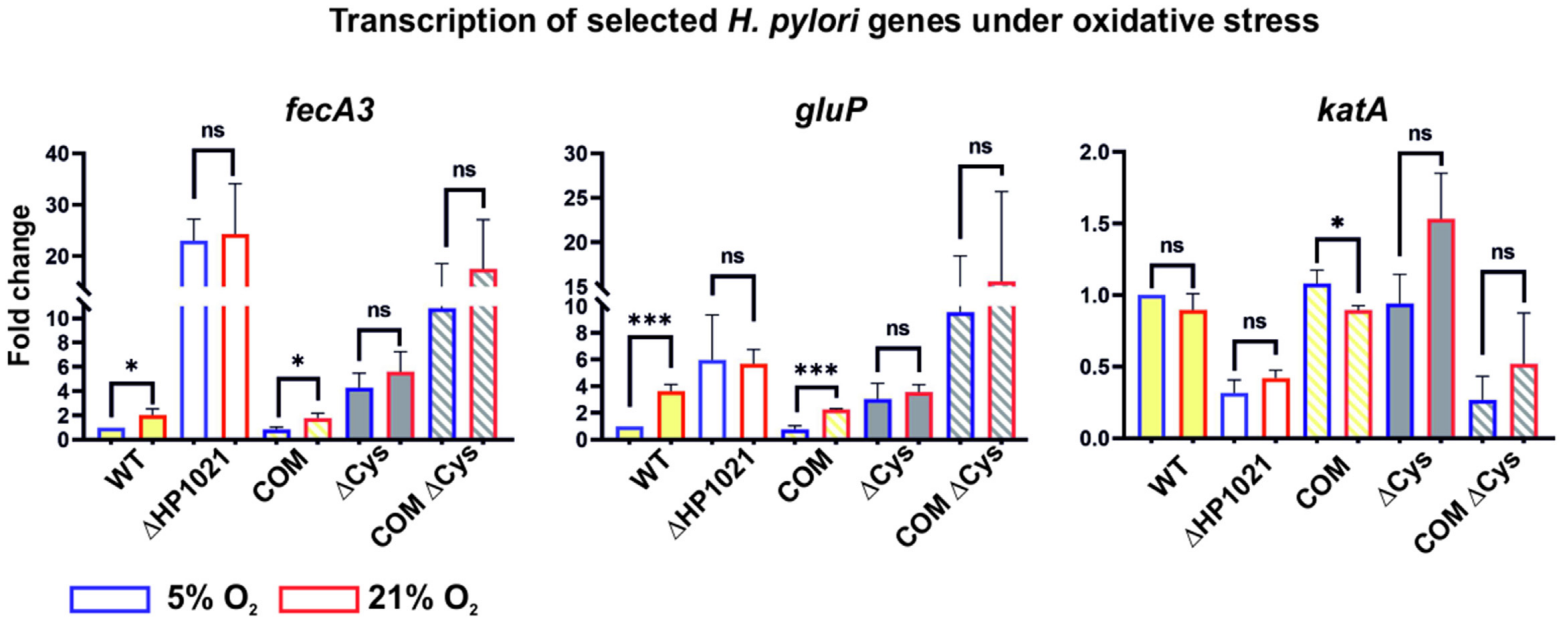
Analysis of the transcription of selected *H. pylori* genes in cells cultured under microaerobic and aerobic stress conditions. Transcription of genes was measured by RT-qPCR in cells from three independent *H. pylori* cultures, and the results are presented as the mean fold change from three analyses. The results of independent RT-qPCR analyses are shown in Supplementary Table S3. Significance values were calculated using a two-tailed Student's *t*-test. ns, not significant; *P < 0.05; **P < 0.01, ***P < 0.001.

Next, using RT-qPCR, we analyzed the transcription of three genes that Pflock and co-workers had shown to be significantly repressed (*gluP, fecA3)* or activated (*katA*) by HP1021 in *H. pylori* 26695. We tested the transcription of these genes in the N6-series strains: WT, ΔHP1021, COM, COM ΔCys and ΔCys under microaerobic and aerobic growth conditions (Figure 9 and Supplementary Table S3). In the ΔHP1021 strain under microaerobic conditions, the transcription of *gluP* and *fecA3* was upregulated (fold change (FC) of 6.0 ± 2.8 and 23.0 ± 3.4, respectively), while the transcription of *katA* in the N6 ΔHP1021 strain was downregulated (FC of 0.3 ± 0.1) compared to that of the N6 WT strain. Thus, the RT-qPCR results confirmed the previous microarray analysis results and indicated that HP1021 controls *gluP*, *fecA3* and *katA* transcription. Next, we analyzed the effect of oxidative stress on the transcription of these genes. In the WT strain, the transcription of *gluP, fecA3* was upregulated compared to their expression in the non-stressed cells (FC of 3.6 ± 0.4, 2.0 ± 0.4). The transcription of *katA* did not significantly change (FC of 0.9 ± 0.1) upon oxidative stress. However, catalase constitutes approximately 4% of the total cell protein (58), which suggests that the *katA* gene is continuously transcribed, and catalase is synthesized in amounts sufficient to ensure enough enzyme is always available to allow the cell to respond to oxidative stress. The transcription of *gluP, fecA3* and *katA* did not change upon oxidative stress in the ΔHP1021 strain compared to the levels under microaerobic conditions. It suggests that HP1021 controls the expression of *gluP* and *fecA3* in response to oxidative stress. The transcription of *katA*, being dependent on HP1021, is not dependent on oxidative stress (see the Discussion section). Analysis of *gluP*, *fecA3*, and *katA* transcription in the complemented COM strain showed that the levels and the stimulation/inhibition pattern of their transcription under microaerobic and aerobe conditions were similar to the levels observed in the wild-type strain. Contrary to the wild-type HP1021 protein in the COM strain, the cysteine-less HP1021 protein could not control the transcription of the analyzed genes in COM ΔCys and ΔCys strains, both under microaerobic conditions or in response to oxidative stress. The different expression of the analyzed genes in both strains, COM ΔCys and ΔCys, producing the cysteine-less HP1021 protein variant might be caused by unknown suppressor mutations induced in the ΔHP1021 strain due to severe homeostasis imbalance in the cells lacking the HP1021 protein. Nonetheless, in the COM strain, the homeostasis was restored by the presence of the wild-type HP1021 protein (i.e., growth, catalase activity and the expression of *fecA*, *gluP* and *katA* genes were similar to the WT strain). Thus, the lack of proper gene expression control in COM ΔCys and ΔCys strains is due to the improper activity of cysteine-less HP1021.

**Figure 10.**
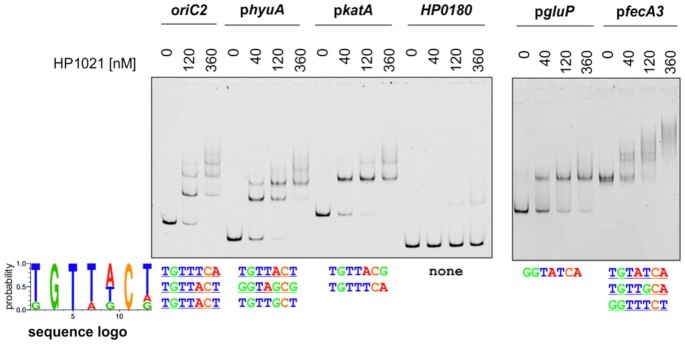
Analysis of the interactions between HP1021 and promoter regions of selected genes. EMSA was performed using the FAM-labeled DNA fragments, which were incubated with the indicated amounts of the TCEP-reduced HP1021 protein. The consensus HP1021 box sequence and the putative HP1021 boxes located in the promoter regions are shown below the gel images. Digital processing was applied equally across the entire image, including controls.

Our results indicated that HP1021 controls the transcription of *gluP* and *fec3*, but no direct interactions between the putative promoter regions of these genes and HP1021 have been shown thus far. Interactions of the HP1021 protein with a few promoter regions (*hyuA* and *katA*) were reported before (27,28). We determined that sequences similar to the consensus sequence of HP1021 box (5’-KGTWDCD-3’, Materials and Methods) were found in each of these putative promoter regions (up to 275 bp upstream or up 99 bp downstream of the translation start site, Figure 10). Thus, we applied EMSA to analyze the binding of HP1021 to *fecA3*, *gluP*, *hyuA* and *katA* promoter regions. As additional controls of the specificity and affinity of interactions, we used *oriC2* and a fragment of the HP0180 gene, i.e. DNA fragments representing specific and high-affinity and unspecific and low-affinity interactions, respectively (Supplementary Figure S9; HP0180, which contains no HP1021 boxes, was also used in SPR as a control of the analysis (Materials and Methods)). We observed that all of the regions but HP0180 were bound by HP1021 (Figure 10). The TCEP-reduced protein bound the promoter regions of *hyuA*, *katA* and *gluP* more readily than the air-oxidized protein, while Zn^2+^ destabilized HP1021-DNA complexes (Supplementary Figure S10; HP1021-*fecA3* interactions were not analyzed). Thus, we confirmed that the redox state and Zn^2+^ affects HP1021 interactions with DNA similarly as observed in HP1021-*oriC2* interactions.

In summary, we have shown that HP1021 controls the transcription of the *gluP, fecA3* and *katA* genes; the control of *fecA3* and *gluP* is redox-dependent. HP1021 likely controls the *katA* transcription in a way independent of the oxidative stress. Further studies are needed to determine the entire regulon of HP1021.

## DISCUSSION

*H. pylori*, living in the human stomach, is exposed to oxidative stress generated by host cells or microbiotic bacteria (59,60). Moreover, it is proposed that *H. pylori* actively shapes the redox state of its niche. For example, nitrosative stress is highly toxic to *H. pylori*; therefore, the bacterium reduces NOS2 expression and, consequently, NO production in the host to enhance its own survival ((39) and references therein). In parallel, *H. pylori* elevates the production of H_2_O_2_ or O_2_, to which it is more adapted. This H_2_O_2_ or O_2_ overproduction leads to ROS-induced mutations in DNA, which increase *H. pylori* genetic variation and adaptability to human hosts, especially during the acute infection phase (39,61,62). It has been recently shown that *H. pylori* senses and migrates towards HClO-rich niches, which are characteristic of inflamed tissues – an efficient source of nutrients, especially iron (63). Therefore, taking into account the constant exposure of *H. pylori* to ROS, it is surprising that the overall repertoire of *H. pylori* proteins to combat oxidative stress is relatively underdeveloped (3,37). One of the *H. pylori* defence system's striking features is the apparent lack of identified redox-switch transcriptional regulators.

However, we propose that the *H. pylori* HP1021 orphan response regulator is, in fact, the redox switch regulator that controls the *H. pylori* response to oxidative stress.

### HP1021 as a redox switch

*H. pylori* HP1021 is characterized by the classical features of a redox switch. HP1021 possesses cysteine residues that are oxidized upon exposure to atmospheric oxygen and diamide (Figures 1, 7 and Supplementary Figure S5). Cysteine oxidation suppresses HP1021 affinity for DNA (Figure 2 and S10A). The presence and redox state of the cysteine residues are also important for metal binding by HP1021, which eventually also affects HP1021 interactions with DNA (Figures 3–6, Supplementary Figures S3, S4 and S10C). However, the molecular mechanism of HP1021 redox control is still unknown, especially because the (i) reactive cysteine residues have not been defined, (ii) metals bound by HP1021 *in vivo* have not been identified and (iii) reactivation mechanism of HP1021 remains unknown.

*In vitro*, oxidized cysteine residues likely form intramolecular disulfide bridges that change the HP1021 protein structure and lower its affinity to DNA. No inter-subunit cysteine bridges were observed *in vivo*, while only a fraction of air- or diamide-oxidized HP1021 formed dimers *in vitro*. The cysteine residues and their oxidation states are both critical for Zn^2+^ binding. However, it is not known which cysteine residues are involved in the redox control of HP1021. Usually, in redox switches, only two (2-Cys-type regulators) and, in some cases, only one (1-Cys-type regulators) of the cysteine residues in a particular molecule are critical for redox sensing (32). For example, of the six cysteine residues in the *E. coli* OxyR redox switch, C199 and C208 are important for OxyR activity control (64); upon oxidation, they form an intramolecular disulfide bond that remodels the protein and changes its DNA-binding activity and interaction with RNA polymerase (RNAP). In *Deinococcus radiodurans* OxyR, only C210 is modified to a sulfenic acid, which changes OxyR activity (65). In HP1021, there are six cysteine residues at conserved positions in the amino acid sequence of the regulator in different *H. pylori* strains (Figure 1 and Supplementary Figure S1). However, only one cysteine residue (C27) is conserved in HP1021 homologs in several species of Campylobacterales (29). This finding suggests that C27 might be involved in the redox control of not only HP1021 but also its homologs in other closely related species, while the involvement of other cysteine residues is more species-specific. Moreover, the localization of cysteine residues in HP1021 is bimodal (C27, C51 and C56 in the N-terminal domain and C216, C238, and C270 in the C-terminal domain), which suggests that the cysteine residues of each domain may be subjected to redox modification and might be important for HP1021 activity. It is tempting to hypothesize that each of the two putative domains, the N-terminal regulatory and C-terminal DNA-binding domains, are redox controlled and eventually cross-talk to control the expression of *H. pylori* genes in response to oxidative stress.

We demonstrated that HP1021 is capable of binding Zn^2+^ (Figures 3 and 4). During our study, we found that Zn^2+^ binding to HP1021 is cysteine- and redox-dependent and consequently affects protein binding to DNA (Figures 5, 6 and Supplementary Figure S10AB). Figure 4 shows that both reduced and oxidized HP1021 wild-type variants bind Zn^2+^; however, the coordination is significantly more efficient in the case of the first variant, indicating the formation of Zn^2+^-protein complex with stoichiometry 1:1. This means that oxidation of the protein (for example, in one domain or specific cysteine residues) decreases affinity for this metal ion, for example, in an allosteric mechanism. An estimation of the dissociation constants based on known Zn^2+^-to-Zincon binding affinity shows that the stability of the Zn^2+^-protein complex drops by at least one order of magnitude, as demonstrated by the *K*_d_ value increase from ∼10^−9^–10^−8^ to 10^−8^–10^−7^ M for the reduced and oxidized forms, respectively. Moreover, the elimination of all cysteine residues from HP1021 caused total metal ion binding disruption regardless of whether the protein had been incubated with TCEP or not. This finding clearly shows that the strength of metal binding greatly depends on the redox status of cysteine residues. Currently, we know neither the exact mechanism of HP1021 oxidation nor the number of oxidized cysteine residues in the protein. Although Zn^2+^ is one of the candidates that bind to HP1021 in a redox-dependent manner, as demonstrated in the cases of other redox switches (32,66), it is not clear whether Zn^2+^ is the native metal ion for *H. pylori*. However, its impact on protein function and obtained affinity constant strongly suggest that it may function as a regulatory metal ion shown in the past for many other Zn^2+^ and DNA-binding proteins in all domains of life (67). In addition to Zn^2+^, Fe^2+^ or Fe-S clusters are often found in redox switches, while Mn^2+^ and Cu^+/2+^ are less commonly found (32–35). We have shown that, although less evident than Zn^2+^, Co^2+^ and Ni^2+^ binds to HP1021 (Figure 3), which destabilizes HP1021-DNA complexes to some extent (Supplementary Figure S4). Therefore, to determine the critical metal ion, additional more accurate metal-oriented investigations are required in the future.

Recombinant HP1021 is reversibly oxidized, i.e., oxidation of HP1021 by atmospheric O_2_ can be reversed by treating HP1021 with the reducing agents DTT or TCEP. In the *H. pylori* cells incubated in 21% O_2_ for 60 min and then switched back to microaerobic conditions, only a partially reduced form of HP1021 was detected. This finding indicates that the cells recovered from oxidative stress; however, it does not allow us to determine the reactivation mechanism of HP1021. In principle, redox sensors can be reactivated by other redox-active proteins, usually thioredoxin or glutaredoxin systems, while the redox sensors that are irreversibly oxidized can be degraded by cellular proteases (e.g. *B. subtilis* PerR oxidized at histidine residues is degraded by LonA) (32,68). The HP1021 protein that is reversibly modified may be reactivated by the thioredoxin system (there is no glutaredoxin system in *H. pylori*). Irreversibly oxidized HP1021 may be subjected to proteolysis, and the resulting reduction in HP1021 would stimulate *de novo* synthesis.

### Redox-dependent transcriptional control mediated by HP1021

At the time when a comparative analysis of genes expressed in the 26695 wild-type and HP1021 knockout strains was conducted by Pflock and co-workers (27), the putative stimulus that mediates HP1021 activity was unknown. Thus, the results apparently depict transcriptional changes at a certain oxidative status. In this work, we analyzed that the N6 and 26695 ΔHP1021 and COM ΔCys mutants were more sensitive to oxidative agents such as diamide, H_2_O_2_ or paraquat than the WT strain; in addition, the 26695 ΔCys mutant strain was more sensitive to diamide and H_2_O_2_ while the N6 ΔCys strain to diamide than the cognate WT strains (Figure 8). The cysteine residues of HP1021 protein were oxidized in *H. pylori* cells under oxidative stress (Figure 7), which is typical for redox sensors. Thus, we proposed that HP1021 responds to oxidative stress. Indeed, we demonstrated that the transcription of selected genes (*gluP* and *fecA3*), which have been previously identified as genes belonging to the HP1021 regulon (27), was induced upon oxidative stress in the WT and COM strains but not in the ΔHP1021, COM ΔCys and ΔCys strains. Thus, these genes are controlled by HP1021 in a redox-dependent manner. HP1021 controlled *katA* but in a redox-independent manner, even though HP1021 *per se* binds *katA* promoter in a redox-dependent manner (Supplementary Figure S10). However, it has been already reported that *katA* expression is relatively high and constant irrespective of redox conditions (58,69,70). It suggests that *H. pylori*, living in an environment of near-constant oxidative stress, produces an excess of catalase to be ready for an immediate detoxifying response either using catalase as an H_2_O_2_ decomposing enzyme or a protector protein quenching harmful oxidants via methionine sulfoxide formation (71,72). Moreover, it was shown that *katA* expression and/or catalase activity are dependent on Fur and/or iron availability (73,74). Thus, catalase expression regulation is probably complex, and additional studies are necessary to reveal the regulatory circuits in details.

Further studies are also required to determine the entire redox controlled HP1021 regulon precisely. However, although relatively limited, our transcription analysis has already indicated additional putative *H. pylori* response mechanisms to oxidative stress that involve the regulation of genes encoding membrane transporters GluP and FecA3. The glucose/galactose transporter GluP has been shown to transport sugars through a Na^+^-dependent mechanism (75–79). Although glucose metabolism is still not fully recognized in *H. pylori*, it has been proposed that both the glucose uptake and usage pathways depend on growth culture conditions (e.g. CO_2_/O_2_ tensions but also glucose availability) (80–83). Interestingly, the GluP transporter has also been shown to act as an efflux pump critical for cell resistance to drugs (amoxicillin and tetracycline) and biofilm formation (84). The transcription of *gluP* depends on a stringent response, and it was proposed that sigma 54-dependent control may be involved (84); however, *gluP* transcription has also been shown to be upregulated upon acid stress (85). It has been previously shown that a stringent response is required for *H. pylori* survival during aerobiosis (86). The transcription of *gluP* is upregulated upon oxidative stress ((83) and Figure 9), and it is constantly upregulated in the HP1021 deletion mutant, regardless of the oxygen level ((27) and Figure 9). This finding suggests that HP1021 either increases glucose uptake to adjust the *H. pylori* metabolism to oxidative stress conditions or activates the efflux of toxic compounds produced during oxidative stress. It should be noted that some other metabolic genes were differentially regulated in the HP1021 deletion mutant (27). Many metabolic genes are also activated upon aerobiosis (83). Therefore, further studies are required to determine whether HP1021 controls metabolic or toxic compound efflux systems in *H. pylori* and the mechanisms involved.

FecA3 is a putative outer membrane Ni^2+^ transport protein (87). *fecA3* transcription is repressed by a pleiotropic NikR regulator in the presence of Ni^2+^ (holo-NikR) as a part of a multistep, hierarchical NikR-dependent transcriptional response that allows *H. pylori* to control nickel homeostasis (10). However, holo-NikR also directly controls iron homeostasis by downregulating the expression of genes encoding iron uptake proteins (*frpb2/frb3/frpb4* and exbB-exbD-tonB) or the ferric uptake regulator Fur (*fur*) (10,11,41); via Fur, holo-NikR also controls iron storage or the activity of iron-dependent enzymes (10,11,41,88). The maintenance of nickel and iron homeostasis is very important since the ions of both are crucial for the activities of the few *H. pylori* enzymes (e.g. urease and hydrogenase), while their excess may lead to cell damage, especially under oxidative stress (6). Our results show that *fecA3* is upregulated upon oxidative stress (2-fold), and the transcription is further increased in the ΔHP1021 mutant strain (approximately 20-fold, irrespectively of the oxidative stress). Since holo-NikR downregulates the expression of genes encoding iron transporters and Fur, it is reasonable to propose that, upon oxidative stress, increased expression of *fecA3* ultimately increases nickel uptake by *H. pylori* cells. An increased Ni^2+^ concentration activates NikR to influence iron homeostasis by decreasing the expression of genes encoding proteins associated with iron uptake and facilitating the Fur-induced increase in the expression of genes encoding iron storage proteins (Pfr) or Fe-dependent enzymes (e.g. SodB or HydA), previously observed to be induced during oxidative stress (88). The role of HP1021 might be to limit excessive nickel uptake during oxidative stress.

Interestingly, HP1021 possibly represents a type of regulator that not only regulates gene expression but also coordinates the bacterial cell cycle with the physiological state of the cell by controlling the initiation of chromosome replication. The HP1021 regulator activity resembles that of the *Mycobacterium tuberculosis* MtrA protein (response regulator of the essential MtrAB two-component system) (89,90). It was proposed that MtrA interactions with key factors of chromosome replication (the promoter region of *dnaA* and *oriC*) regulate *M. tuberculosis* cell cycle progression and contributes to *M. tuberculosis* survival in macrophages. Thus, further comprehensive studies on the HP1021 regulator may help to characterize dual role regulators, such as MtrA, which are pivotal for bacterial survival under different, not always optimal, growth conditions.

In conclusion, we propose that HP1021 is a novel redox-sensor regulator that enhances *H. pylori* in combatting oxidative stress. Further research should help to determine the exact molecular mechanism of redox-dependent HP1021 activity regulation and the HP1021 regulon. In a more general context, characterization of the HP1021 regulon will help to determine the oxidative stress modulon that has not been characterized at a satisfactory level.

## DATA AVAILABILITY

Data supporting the findings of this manuscript will be available from the corresponding author after publication upon reasonable request.

## Supplementary Material

gkab440_Supplemental_File

## References

[1] Sgouras D. , Tegtmeyer N., Wessler S. Activity and functional importance of *Helicobacter pylori* virulence factors. Advances in Experimental Medicine and Biology. 2019; 1149:NY Springer 35–56. 31016624 10.1007/5584_2019_358

[2] Ansari S. , Yamaoka Y. Survival of *Helicobacter pylori* in gastric acidic territory. Helicobacter. 2017; 22:e12386. 10.1111/hel.12386 PMC5851894 28402047

[3] Flint A. , Stintzi A., Saraiva L.M. Oxidative and nitrosative stress defences of *Helicobacter* and *Campylobacter* species that counteract mammalian immunity. FEMS Microbiol. Rev. 2016; 40:938–960. 28201757 10.1093/femsre/fuw025 PMC5091033

[4] Ulrich L.E. , Zhulin I.B. The MiST2 database: a comprehensive genomics resource on microbial signal transduction. Nucleic Acids Res. 2010; 38:D401–D407. 19900966 10.1093/nar/gkp940 PMC2808908

[5] Danielli A. , Amore G., Scarlato V. Built shallow to maintain homeostasis and persistent infection: insight into the transcriptional regulatory network of the gastric human pathogen *Helicobacter pylori*. PLoS Pathog. 2010; 6:e1000938. 20548942 10.1371/journal.ppat.1000938 PMC2883586

[6] Haley K.P. , Gaddy J.A. Metalloregulation of *Helicobacter pylori* physiology and pathogenesis. Front. Microbiol. 2015; 6:911. 26388855 10.3389/fmicb.2015.00911 PMC4557348

[7] Johnson K.S. , Ottemann K.M. Colonization, localization, and inflammation: the roles of *H. pylori* chemotaxis *in vivo*. Curr. Opin. Microbiol. 2018; 41:51–57. 29202336 10.1016/j.mib.2017.11.019 PMC5862749

[8] Jones M.D. , Li Y., Zamble D.B. Acid-responsive activity of the *Helicobacter pylori* metalloregulator NikR. Proc. Natl. Acad. Sci. 2018; 115:8966–8971. 30126985 10.1073/pnas.1808393115 PMC6130374

[9] Pelliciari S. , Vannini A., Roncarati D., Danielli A. The allosteric behavior of Fur mediates oxidative stress signal transduction in *Helicobacter pylori*. Front. Microbiol. 2015; 6:840. 26347726 10.3389/fmicb.2015.00840 PMC4541418

[10] Muller C. , Bahlawane C., Aubert S., Delay C.M., Schauer K., Michaud-Soret I., De Reuse H. Hierarchical regulation of the NikR-mediated nickel response in *Helicobacter pylori*. Nucleic Acids Res. 2011; 39:7564–7575. 21666253 10.1093/nar/gkr460 PMC3177205

[11] Vannini A. , Pinatel E., Costantini P.E., Pelliciari S., Roncarati D., Puccio S., De Bellis G., Peano C., Danielli A. Comprehensive mapping of the *Helicobacter pylori* NikR regulon provides new insights in bacterial nickel responses. Sci. Rep. 2017; 7:45458. 28393877 10.1038/srep45458 PMC5385501

[12] Tsang J. , Hirano T., Hoover T.R., McMurry J.L. *Helicobacter pylori* FlhA binds the sensor kinase and flagellar gene regulatory protein FlgS with high affinity. J. Bacteriol. 2015; 197:1886–1892. 25802298 10.1128/JB.02610-14 PMC4420913

[13] Spohn G. , Scarlato V. Motility of *Helicobacter pylori* is coordinately regulated by the transcriptional activator FlgR, an NtrC homolog. J. Bacteriol. 1999; 181:593–599. 9882675 10.1128/jb.181.2.593-599.1999 PMC93415

[14] Niehus E. , Gressmann H., Ye F., Schlapbach R., Dehio M., Dehio C., Stack A., Meyer T.F., Suerbaum S., Josenhans C. Genome-wide analysis of transcriptional hierarchy and feedback regulation in the flagellar system of *Helicobacter pylori*. Mol. Microbiol. 2004; 52:947–961. 15130117 10.1111/j.1365-2958.2004.04006.x

[15] Pflock M. , Kennard S., Finsterer N., Beier D. Acid-responsive gene regulation in the human pathogen *Helicobacter pylori*. J. Biotechnol. 2006; 126:52–60. 16713649 10.1016/j.jbiotec.2006.03.045

[16] Pflock M. , Kennard S., Delany I., Scarlato V., Beier D. Acid-induced activation of the urease promoters is mediated directly by the ArsRS two-component system of *Helicobacter pylori*. Infect. Immun. 2005; 73:6437–6445. 16177315 10.1128/IAI.73.10.6437-6445.2005 PMC1230922

[17] Loh J.T. , Gupta S.S., Friedman D.B., Krężel A.M., Cover T.L. Analysis of protein expression regulated by the *Helicobacter pylori* ArsRS two-component signal transduction system. J. Bacteriol. 2010; 192:2034–2043. 20154125 10.1128/JB.01703-08 PMC2849440

[18] Loh J.T. , Shum M. V., Jossart S.D.R., Campbell A.M., Sawhney N., McDonald W.H., Scholz M.B., McClain M.S., Forsyth M.H., Cover T.L Delineation of the pH-responsive regulon controlled by the *Helicobacter pylori* ArsRS two- component system. Infect. Immun. 2021; 89:e00597-20. 33526561 10.1128/IAI.00597-20 PMC8090972

[19] Waidner B. , Melchers K., Stahler F.N., Kist M., Bereswill S. The *Helicobacter pylori* CrdRS two-component regulation system (HP1364/HP1365) is required for copper-mediated induction of the copper resistance determinant CrdA. J. Bacteriol. 2005; 187:4683–4688. 15968080 10.1128/JB.187.13.4683-4688.2005 PMC1151771

[20] Hung C.-L. , Cheng H.-H., Hsieh W.-C., Tsai Z.T.-Y., Tsai H.-K., Chu C.-H., Hsieh W.-P., Chen Y.-F., Tsou Y., Lai C.-H. et al. The CrdRS two-component system in *Helicobacter pylori* responds to nitrosative stress. Mol. Microbiol. 2015; 97:1128–1141. 26082024 10.1111/mmi.13089

[21] Beier D. , Frank R. Molecular characterization of two-component systems of *Helicobacter pylori*. J. Bacteriol. 2000; 182:2068–2076. 10735847 10.1128/jb.182.8.2068-2076.2000 PMC111253

[22] Delany I. , Spohn G., Rappuoli R., Scarlato V. Growth phase-dependent regulation of target gene promoters for binding of the essential orphan response regulator HP1043 of *Helicobacter pylori*. J. Bacteriol. 2002; 184:4800–4810. 12169605 10.1128/JB.184.17.4800-4810.2002 PMC135297

[23] Pelliciari S. , Pinatel E., Vannini A., Peano C., Puccio S., De Bellis G., Danielli A., Scarlato V., Roncarati D. Insight into the essential role of the *Helicobacter pylori* HP1043 orphan response regulator: genome-wide identification and characterization of the DNA-binding sites. Sci. Rep. 2017; 7:41063. 28112213 10.1038/srep41063 PMC5253667

[24] Olekhnovich I.N. , Vitko S., Chertihin O., Hontecillas R., Viladomiu M., Bassaganya-Riera J., Hoffman P.S. Mutations to essential orphan response regulator HP1043 of *Helicobacter pylori* result in growth-stage regulatory defects. Infect. Immun. 2013; 81:1439–1449. 23429531 10.1128/IAI.01193-12 PMC3647984

[25] Vannini A. , Roncarati D., Danielli A. The *cag*-pathogenicity island encoded CncR1 sRNA oppositely modulates *Helicobacter pylori* motility and adhesion to host cells. Cell. Mol. Life Sci. 2016; 73:3151–3168. 26863876 10.1007/s00018-016-2151-z PMC11108448

[26] McDaniel T.K. , Dewalt K.C., Salama N.R., Falkow S. New approaches for validation of lethal phenotypes and genetic reversion in *Helicobacter pylori*. Helicobacter. 2001; 6:15–23. 11328361 10.1046/j.1523-5378.2001.00001.x

[27] Pflock M. , Bathon M., Schär J., Müller S., Mollenkopf H., Meyer T.F., Beier D. The orphan response regulator HP1021 of *Helicobacter pylori* regulates transcription of a gene cluster presumably involved in acetone metabolism. J. Bacteriol. 2007; 189:2339–2349. 17220217 10.1128/JB.01827-06 PMC1899378

[28] Donczew R. , Makowski Ł., Jaworski P., Bezulska M., Nowaczyk M., Zakrzewska-Czerwińska J., Zawilak-Pawlik A. The atypical response regulator HP1021 controls formation of the *Helicobacter pylori* replication initiation complex. Mol. Microbiol. 2015; 95:297–312. 25402746 10.1111/mmi.12866

[29] Schär J. , Sickmann A., Beier D. Phosphorylation-independent activity of atypical response regulators of *Helicobacter pylori*. J. Bacteriol. 2005; 187:3100–3109. 15838037 10.1128/JB.187.9.3100-3109.2005 PMC1082831

[30] Imlay J.A. Cellular defenses against superoxide and hydrogen peroxide. Annu. Rev. Biochem. 2008; 77:755–776. 18173371 10.1146/annurev.biochem.77.061606.161055 PMC3057177

[31] Lee S.J. , Kim D.-G., Lee K.-Y., Koo J.S., Lee B.-J. Regulatory mechanisms of thiol-based redox sensors: lessons learned from structural studies on prokaryotic redox sensors. Arch. Pharm. Res. 2018; 41:583–593. 29777359 10.1007/s12272-018-1036-0

[32] Hillion M. , Antelmann H. Thiol-based redox switches in prokaryotes. Biol. Chem. 2015; 396:415–444. 25720121 10.1515/hsz-2015-0102 PMC4438307

[33] Arnesano F. , Banci L., Bertini I., Mangani S., Thompsett A.R. A redox switch in CopC: an intriguing copper trafficking protein that binds copper(I) and copper(II) at different sites. Proc. Natl. Acad. Sci. USA. 2003; 100:3814–3819. 12651950 10.1073/pnas.0636904100 PMC153004

[34] Outten F.W. , Theil E.C. Iron-based redox switches in biology. Antioxid. Redox Signal. 2009; 11:1029–1046. 19021503 10.1089/ars.2008.2296 PMC2842161

[35] Herbig A.F. , Helmann J.D. Roles of metal ions and hydrogen peroxide in modulating the interaction of the *Bacillus subtilis* PerR peroxide regulon repressor with operator DNA. Mol. Microbiol. 2001; 41:849–859. 11532148 10.1046/j.1365-2958.2001.02543.x

[36] Wang G. , Alamuri P., Maier R.J. The diverse antioxidant systems of *Helicobacter pylori*. Mol. Microbiol. 2006; 61:847–860. 16879643 10.1111/j.1365-2958.2006.05302.x

[37] Stent A. , Every A.L., Sutton P. *Helicobacter pylori* defense against oxidative attack. Am. J. Physiol. Liver Physiol. 2012; 302:G579–G587. 10.1152/ajpgi.00495.2011 22194421

[38] Collins K.D. , Hu S., Grasberger H., Kao J.Y., Ottemann K.M. Chemotaxis allows bacteria to overcome host-generated reactive oxygen species that constrain gland colonization. Infect. Immun. 2018; 86:e00878-17. 29507083 10.1128/IAI.00878-17 PMC5913845

[39] Gobert A.P. , Wilson K.T. The immune battle against *Helicobacter pylori* infection: no offense. Trends Microbiol. 2016; 24:366–376. 26916789 10.1016/j.tim.2016.02.005 PMC4841705

[40] Krüger N.-J.J. , Knüver M.-T.T., Zawilak-Pawlik A., Appel B., Stingl K. Genetic diversity as consequence of a microaerobic and neutrophilic lifestyle. PLoS Pathog. 2016; 12:e1005626. 27166672 10.1371/journal.ppat.1005626 PMC4864210

[41] Contreras M. , Thiberge J.-M., Mandrand-Berthelot M.-A., Labigne A. Characterization of the roles of NikR, a nickel-responsive pleiotropic autoregulator of *Helicobacter pylori*. Mol. Microbiol. 2003; 49:947–963. 12890020 10.1046/j.1365-2958.2003.03621.x

[42] Kocyła A. , Pomorski A., Krężel A. Molar absorption coefficients and stability constants of Zincon metal complexes for determination of metal ions and bioinorganic applications. J. Inorg. Biochem. 2017; 176:53–65. 28863280 10.1016/j.jinorgbio.2017.08.006

[43] Scheuermann T.H. , Brautigam C.A. High-precision, automated integration of multiple isothermal titration calorimetric thermograms: New features of NITPIC. Methods. 2015; 76:87–98. 25524420 10.1016/j.ymeth.2014.11.024 PMC4380771

[44] Houtman J.C.D. , Brown P.H., Bowden B., Yamaguchi H., Appella E., Samelson L.E., Schuck P. Studying multisite binary and ternary protein interactions by global analysis of isothermal titration calorimetry data in SEDPHAT: Application to adaptor protein complexes in cell signaling. Protein Sci. 2007; 16:30–42. 17192587 10.1110/ps.062558507 PMC1794685

[45] Majka J. , Speck C. Analysis of protein–DNA interactions using surface plasmon resonance. Adv. Biochem. Eng. /Biotechnol. 2007; 104:13–36. 17290817

[46] Zawilak-Pawlik A. , Donczew R., Szafrański S., Mackiewicz P., Terradot L., Zakrzewska-Czerwińska J. DiaA/HobA and DnaA: a pair of proteins co-evolved to cooperate during bacterial orisome assembly. J. Mol. Biol. 2011; 408:238–251. 21354425 10.1016/j.jmb.2011.02.045

[47] Denoncin K. , Nicolaes V., Cho S.-H., Leverrier P., Collet J.-F. Protein disulfide bond formation in the periplasm: determination of the in vivo redox state of cysteine residues. Methods in Molecular Biology. 2013; 966:Clifton, NJ 325–336. 23299744 10.1007/978-1-62703-245-2_20

[48] Fratelli M. , Demol H., Puype M., Casagrande S., Eberini I., Salmona M., Bonetto V., Mengozzi M., Duffieux F., Miclet E. et al. Identification by redox proteomics of glutathionylated proteins in oxidatively stressed human T lymphocytes. Proc. Natl. Acad. Sci. U.S.A. 2002; 99:3505–3510. 11904414 10.1073/pnas.052592699 PMC122553

[49] Kelley L.A. , Mezulis S., Yates C.M., Wass M.N., Sternberg M.J.E. The Phyre2 web portal for protein modeling, prediction and analysis. Nat. Protoc. 2015; 10:845–858. 25950237 10.1038/nprot.2015.053 PMC5298202

[50] Giles N.M. , Watts A.B., Giles G.I., Fry F.H., Littlechild J.A., Jacob C. Metal and redox modulation of cysteine protein function. Chem. Biol. 2003; 10:677–693. 12954327 10.1016/s1074-5521(03)00174-1

[51] Waldron K.J. , Robinson N.J. How do bacterial cells ensure that metalloproteins get the correct metal. Nat. Rev. Microbiol. 2009; 7:25–35. 19079350 10.1038/nrmicro2057

[52] Quinn C.F. , Carpenter M.C., Croteau M.L., Wilcox D.E. Isothermal titration calorimetry measurements of metal ions binding to proteins. Methods in Enzymology. 2016; 567:Academic Press Inc 3–21. 26794348 10.1016/bs.mie.2015.08.021

[53] Krężel A. , Latajka R., Bujacz G.D., Bal W. Coordination properties of tris(2-carboxyethyl)phosphine, a newly introduced thiol reductant, and its oxide. Inorg. Chem. 2003; 42:1994–2003. 12639134 10.1021/ic025969y

[54] Kluska K. , Adamczyk J., Krężel A. Metal binding properties of zinc fingers with a naturally altered metal binding site. Metallomics. 2018; 10:248–263. 29230465 10.1039/c7mt00256d

[55] Barber-Zucker S. , Shaanan B., Zarivach R. Transition metal binding selectivity in proteins and its correlation with the phylogenomic classification of the cation diffusion facilitator protein family. Sci. Rep. 2017; 7:16381. 29180655 10.1038/s41598-017-16777-5 PMC5703985

[56] Ole Leichert L.I. , Scharf C., Hecker M. Global characterization of disulfide stress in *Bacillus subtilis*. J. Bacteriol. 2003; 185:1967–1975. 12618461 10.1128/JB.185.6.1967-1975.2003 PMC150141

[57] Kallifidas D. , Thomas D., Doughty P., Paget M.S.B The σR regulon of *Streptomyces coelicolor* A3(2) reveals a key role in protein quality control during disulphide stress. Microbiology. 2010; 156:1661–1672. 20185507 10.1099/mic.0.037804-0

[58] Mahawar M. , Tran V.L., Sharp J.S., Maier R.J. Synergistic roles of *Helicobacter pylori* methionine sulfoxide reductase and GroEl in repairing oxidant-damaged catalase. J. Biol. Chem. 2011; 286:19159–19169. 21460217 10.1074/jbc.M111.223677 PMC3099729

[59] Imlay J.A. Where in the world do bacteria experience oxidative stress. Environ. Microbiol. 2019; 21:521–530. 30307099 10.1111/1462-2920.14445 PMC7301649

[60] Pérez S. , Taléns-Visconti R., Rius-Pérez S., Finamor I., Sastre J. Redox signaling in the gastrointestinal tract. *Free**Radic*. Biol. Med. 2017; 104:75–103. 10.1016/j.freeradbiomed.2016.12.048 28062361

[61] Gobert A.P. , Wilson K.T. Polyamine- and NADPH-dependent generation of ROS during *Helicobacter pylori* infection: a blessing in disguise. *Free**Radic*. Biol. Med. 2017; 105:16–27. 10.1016/j.freeradbiomed.2016.09.024 PMC5366100 27682363

[62] Linz B. , Windsor H.M., McGraw J.J., Hansen L.M., Gajewski J.P., Tomsho L.P., Hake C.M., Solnick J. V., Schuster S.C., Marshall B.J. A mutation burst during the acute phase of *Helicobacter pylori* infection in humans and rhesus macaques. Nat. Commun. 2014; 5:4165. 24924186 10.1038/ncomms5165

[63] Perkins A. , Tudorica D.A., Amieva M.R., James Remington S., Guillemin K. *Helicobacter pylori* senses bleach (HOCl) as a chemoattractant using a cytosolic chemoreceptor. PLoS Biol. 2019; 17:e3000395. 31465435 10.1371/journal.pbio.3000395 PMC6715182

[64] Zheng M. , Åslund F., Storz G. Activation of the OxyR Transcription Factor by Reversible Disulfide Bond Formation. Science. 1998; 279:1718–1721. 9497290 10.1126/science.279.5357.1718

[65] Chen H. , Xu G., Zhao Y., Tian B., Lu H., Yu X., Xu Z., Ying N., Hu S., Hua Y. A novel OxyR sensor and regulator of hydrogen peroxide stress with one cysteine residue in *Deinococcus radiodurans*. PLoS One. 2008; 3:e1602. 18270589 10.1371/journal.pone.0001602 PMC2225504

[66] Andreini C. , Banci L., Bertini I., Rosato A. Zinc through the three domains of life. J. Proteome Res. 2006; 5:3173–3178. 17081069 10.1021/pr0603699

[67] Kocyła A. , Tran J.B., Krężel A. Galvanization of protein–protein interactions in a dynamic zinc interactome. Trends Biochem. Sci. 2021; 46:64–79. 32958327 10.1016/j.tibs.2020.08.011

[68] Ahn B.E. , Baker T.A. Oxidization without substrate unfolding triggers proteolysis of the peroxide-sensor, PerR. Proc. Natl. Acad. Sci. U.S.A. 2016; 113:E23–E31. 26677871 10.1073/pnas.1522687112 PMC4711837

[69] Huang C.-H. , Chiou S.-H. Proteomic analysis of upregulated proteins in *Helicobacter pylori* under oxidative stress induced by hydrogen peroxide. Kaohsiung J. Med. Sci. 2011; 27:544–553. 22208537 10.1016/j.kjms.2011.06.019 PMC11916125

[70] Wang G. , Olczak A., Forsberg L.S., Maier R.J. Oxidative stress-induced peptidoglycan deacetylase in *Helicobacter pylori*. J. Biol. Chem. 2009; 284:6790–6800. 19147492 10.1074/jbc.M808071200 PMC2652260

[71] Benoit S.L. , Maier R.J. *Helicobacter* catalase devoid of catalytic activity protects the bacterium against oxidative stress. J. Biol. Chem. 2016; 291:23366–23373. 27605666 10.1074/jbc.M116.747881 PMC5095394

[72] Harris A.G. , Hinds F.E., Beckhouse A.G., Kolesnikow T., Hazell S.L. Resistance to hydrogen peroxide in *Helicobacter pylori*: role of catalase (KatA) and Fur, and functional analysis of a novel gene product designated ‘KatA-associated protein’, KapA (HP0874). Microbiology. 2002; 148:3813–3825. 12480885 10.1099/00221287-148-12-3813

[73] Ernst F.D. , Bereswill S., Waidner B., Stoof J., Mäder U., Kusters J.G., Kuipers E.J., Kist M., van Vliet A.H.M., Homuth G. Transcriptional profiling of *Helicobacter pylori* Fur- and iron-regulated gene expression. Microbiology. 2005; 151:533–546. 15699202 10.1099/mic.0.27404-0

[74] Danielli A. , Roncarati D., Delany I., Chiarini V., Rappuoli R., Scarlato V. *In vivo* dissection of the *Helicobacter pylori* fur regulatory circuit by genome-wide location analysis. J. Bacteriol. 2006; 188:4654–4662. 16788174 10.1128/JB.00120-06 PMC1483005

[75] Mendz G.L. , Burns B.P., Hazell S.L. Characterisation of glucose transport in *Helicobacter pylori*. BBA - Gen. Subj. 1995; 1244:269–276. 10.1016/0304-4165(95)00018-7 7599143

[76] Mendz G.L. , Hazell S.L., Burns B.P. Glucose utilization and lactate production by *Helicobacter pylori*. J. Gen. Microbiol. 1993; 139:3023–3028. 8126428 10.1099/00221287-139-12-3023

[77] Psakis G. , Saidijam M., Shibayama K., Polaczek J., Bettaney K.E., Baldwin J.M., Baldwin S.A., Hope R., Essen L.O., Essenberg R.C. et al. The sodium-dependent D-glucose transport protein of *Helicobacter pylori*. Mol. Microbiol. 2009; 71:391–403. 19161491 10.1111/j.1365-2958.2008.06535.x

[78] Bury-Moné S. , Mendz G.L., Ball G.E., Thibonnier M., Stingl K., Ecobichon C., Avé P., Huerre M., Labigne A., Thiberge J.-M. et al. Roles of alpha and beta carbonic anhydrases of *Helicobacter pylori* in the urease-dependent response to acidity and in colonization of the murine gastric mucosa. Infect. Immun. 2008; 76:497–509. 18025096 10.1128/IAI.00993-07 PMC2223474

[79] Som S. , De A., Banik G.D., Maity A., Ghosh C., Pal M., Daschakraborty S.B., Chaudhuri S., Jana S., Pradhan M. Mechanisms linking metabolism of *Helicobacter pylori* to ^18^O and ^13^C-isotopes of human breath CO_2_. Sci. Rep. 2015; 5:10936. 26039789 10.1038/srep10936 PMC4454186

[80] Marais A. , Mendz G.L., Hazell S.L., Mégraud F. Metabolism and genetics of *Helicobacter pylori*: the genome era. Microbiol. Mol. Biol. Rev. 1999; 63:642–674. 10477311 10.1128/mmbr.63.3.642-674.1999 PMC103749

[81] Hazell S.L. , Mendz G.L. How *Helicobacter pylori* works: an overview of the metabolism of *Helicobacter pylori*. Helicobacter. 1997; 2:1–12. 9432315 10.1111/j.1523-5378.1997.tb00050.x

[82] Park S. , Ko A., Lee N. Stimulation of growth of the human gastric pathogen *Helicobacter pylori* by atmospheric level of oxygen under high carbon dioxide tension. BMC Microbiol. 2011; 11:96. 21569333 10.1186/1471-2180-11-96 PMC3110553

[83] Park S.A. , Lee N.G. Global regulation of gene expression in the human gastric pathogen *Helicobacter pylori* in response to aerobic oxygen tension under a high carbon dioxide level. J. Microbiol. Biotechnol. 2013; 23:451–458. 23568198 10.4014/jmb.1209.09064

[84] Ge X. , Cai Y., Chen Z., Gao S., Geng X., Li Y., Li Y., Jia J., Sun Y. Bifunctional enzyme SpoT is involved in biofilm formation of *Helicobacter pylori* with multidrug resistance by upregulating efflux pump Hp1174 (*gluP*). Antimicrob. Agents Chemother. 2018; 62:e00957-18. 30181372 10.1128/AAC.00957-18 PMC6201075

[85] Wen Y. , Marcus E.A., Matrubutham U., Gleeson M.A., Scott D.R., Sachs G. Acid-adaptive genes of *Helicobacter pylori*. Infect. Immun. 2003; 71:5921–5939. 14500513 10.1128/IAI.71.10.5921-5939.2003 PMC201084

[86] Mouery K. , Rader B.A., Gaynor E.C., Guillemin K. The stringent response is required for *Helicobacter pylori* survival of stationary phase, exposure to acid, and aerobic shock. J. Bacteriol. 2006; 188:5494–5500. 16855239 10.1128/JB.00366-06 PMC1540029

[87] Ernst F.D. , Stoof J., Horrevoets W.M., Kuipers E.J., Kusters J.G., van Vliet A.H.M. NikR mediates nickel-responsive transcriptional repression of the *Helicobacter pylori* outer membrane proteins FecA3 (HP1400) and FrpB4 (HP1512). Infect. Immun. 2006; 74:6821–6828. 17015456 10.1128/IAI.01196-06 PMC1698083

[88] Pelliciari S. , Vannini A., Roncarati D., Danielli A. The allosteric behavior of Fur mediates oxidative stress signal transduction in *Helicobacter pylori*. Front. Microbiol. 2015; 6:840. 26347726 10.3389/fmicb.2015.00840 PMC4541418

[89] Fol M. , Chauhan A., Nair N.K., Maloney E., Moomey M., Jagannath C., Madiraju M.V.V.S., Rajagopalan M Modulation of *Mycobacterium tuberculosis* proliferation by MtrA, an essential two-component response regulator. Mol. Microbiol. 2006; 60:643–657. 16629667 10.1111/j.1365-2958.2006.05137.x

[90] Purushotham G. , Sarva K.B., Blaszczyk E., Rajagopalan M., Madiraju M.V. *Mycobacterium tuberculosis oriC* sequestration by MtrA response regulator. Mol. Microbiol. 2015; 98:586–604. 26207528 10.1111/mmi.13144 PMC4700885

