# Supplementary material for: HP1021 is a redox switch protein identified in Helicobacter pylori
Source: Nucleic Acids Res. 2021 Jun 17;49(12):6863–79. doi: 10.1093/nar/gkab440 (PMC8266642; doi:10.1093/nar/gkab440)
Supplement: gkab440_Supplemental_File [file gkab440_supplemental_file.pdf]

Supplementary Data for

HP1021 is a redox switch protein identified in *Helicobacter pylori*

Piotr Szczepanowski[1]†, Mateusz Noszka[1]†, Dorota Żyła-Uklejewicz[1], Fabian Pikuła1, Malgorzata Nowaczyk-Cieszewska[1], Artur Krężel[2], Kerstin Stingl[3], Anna Zawilak-Pawlik[1*]

[1] Department of Microbiology, Hirszfeld Institute of Immunology and Experimental Therapy, Polish Academy of Sciences, Wrocław, 53-114, Poland

[2] Department of Chemical Biology, Faculty of Biotechnology, University of Wrocław, Wrocław, 50-383, Poland

[3] Department of Biological Safety, National Reference Laboratory for *Campylobacter,* German Federal Institute for Risk Assessment, Berlin, 12277, Germany

* To whom correspondence should be addressed: Anna Pawlik, Tel +48 71 3709949; Fax: +48 713372171; Email: anna.pawlik@hirszfeld.pl

† These authors contributed equally to this work

SUPPLEMENTARY INFORMATION TEXT

SUPPLEMENTARY MATERIALS AND METHODS

Construction of plasmids expressing recombinant wild-type and mutant HP1021 variants

pET28Strep was chosen as an expression vector for the synthesis of the wild-type HP1021 and cysteine-less HP1021ΔCys variant of the *H. pylori* HP1021 protein. pET28Strep is a modified pET28a(+) vector (Supplementary Table S1) that produces strep-tagged proteins (WSHPQFEK, Strep-tag II) at the N-terminus. The *HP1021* gene (1-897 bp) was amplified with primer pairs P3-P4 using *H. pylori* 26695 genomic DNA as a template. The PCR products were digested with BamHI/XhoI and cloned into BamHI/XhoI sites of pET28Strep to generate pETStrepHP1021 (Supplementary Table S1). To construct the pET28StrepHP1021ΔCys plasmid, for use in synthesizing the cysteine-less HP1021ΔCys protein, seven PCR fragments were amplified using the pET28StrepHP1021 plasmid as a template and pairs of primers that were designed to introduce point mutations into cysteine residue codons to change them into alanine codons and to provide short homologous sequences that enabled the PCR products to be hybridized and used as DNA templates in the second round of PCR (Supplementary Figure S2 and Supplementary Table S2). The fragments were amplified as follows: fragment 1 (P5-P7), fragment 2 (P6-P9), fragment 3 (P8-P16), fragment 4 (P5-P11), fragment 5 (P10-P13), fragment 6 (P12-P15) and fragment 7 (P14-P16). All PCR products were purified using an agarose gel and used as templates for DNA amplification. DNA fragments 1-3 were amplified using the P5-P16 primer pair to generate a 965-bp PCR *HP1021NΔCys* fusion product in which C27, C51 and C56 were mutated to Ala residues. DNA fragments 4-7 were amplified by the P5-P16 primer pair to generate a 965-bp PCR *HP1021CΔCys* fusion product in which C216, C238 and C270 were mutated to alanine residues. *HP1021NΔCys* and *HP1021CΔCys* were digested with BamHI/XhoI and cloned into the BamHI/XhoI sites of pET28Strep, generating pETStrepHP1021*NΔCys* and pETStrepHP1021*CΔCys* (Supplementary Table S1). In the final step, the 621-bp DNA fragment was excised from pETStrepHP1021*NΔCys* with BamHI/MunI and cloned into a 5632-bp fragment of pETStrepHP1021*CΔCys* digested with the same restriction enzymes, generating the final pET28StrepHP1021ΔCys vector.

Construction of the *H. pylori* mutant strains

H. pylori mutants were constructed using a homologous recombination approach. *H. pylori* cells were transformed with purified plasmids by natural transformation. For the selection of *H. pylori* transformants (after three to five days of growth), kanamycin and/or chloramphenicol was added to the medium at concentrations of 15 μg ml^{-1} and 8 μg ml^{-1}, respectively.

<u>*H. pylori* ΔHP1021</u>

The *H. pylori* N6 ΔHP1021 mutant with the entire HP1021 gene deleted from the chromosome was constructed as described previously for the 26695 strain using the pTZ57R/TΔHP1021 plasmid ((1) and Supplementary Figure S11B). The *H. pylori* 26695 ΔHP1021 strain was constructed *de novo* for the purpose of this work to minimize the number of passages in the construction workflow of derivative strains (COM and COM ΔCys).

<u>*H. pylori* COM</u>

The *H. pylori* HP1021 complementation mutant strains (N6 COM, 26695 COM) were constructed as follows (Supplementary Figure S11C). The region upstream of *HP1021* and the *HP1021* gene were amplified by PCR using the P81-P63 primer pair, while the downstream region flanking *HP1021* was amplified by PCR using the P66-P82 primer pair; both regions were amplified using *H. pylori* 26695 genomic DNA as a template. The *cat* cassette was amplified using the P64-

P65 primer pair and *H. pylori* N6 ΔhtrA/htrA$_{N6}$ genomic DNA (2) as a template. The resulting fragments were purified on an agarose gel and used as a template to produce a joint DNA fragment using the P81-P82 primer pair. The resulting PCR fragment HP1021-*cat* was blunt-end cloned into the SmaI-linearised pUC18 cloning vector generating pUC18HP1021com. The obtained plasmid was subsequently used to transform *H. pylori* N6 and 26695 strains. The transformants were selected by plating on Columbia blood agar plates supplemented with chloramphenicol. The allelic exchange was verified by PCR using the P57-P58 primers, which were homologous to the chromosomal regions external to the designed recombination sites, and by Western blot analysis using a rabbit polyclonal anti-HP1021 antibody.

H. pylori ΔCys

H. pylori ΔCys mutants (N6 ΔCys and 26695 ΔCys), which synthesize the cysteine-less HP1021ΔCys protein variant, were constructed as follows (Supplementary Figure S11E). The 3' region of *HP1020* and the 5' region of HP1022 were amplified by PCR using two pairs of primers P49-P60 and P61-P50, respectively, and *H. pylori* 26695 ΔHP1021 genomic DNA as a template. HP1021ΔCys was amplified using the P59-P62 primer pair and the pET28StrepHP1021ΔCys vector as a template. The resulting fragments were purified using an agarose gel and used as a template to produce a joint DNA fragment using the P49-P50 primer pair. The resulting PCR fragment was subsequently cloned into the pGEM-T Easy vector to yield pGEM-T Easy HP1021ΔCys. *H. pylori* N6 and 26695 strains were transformed with pGEM-T Easy HP1021ΔCys, and transformants were selected by plating on Columbia blood agar plates supplemented with kanamycin. The allelic exchange was verified by PCR using the P57-P58 primer pair homologous to chromosomal regions external to the designed recombination sites. The *HP1021* was amplified using P79-P80 primers and sequenced to verify the correct recombination and exchange of the codons for cysteine to the codons for alanine. The synthesis of HP1021ΔCys was verified by Western blot analysis using a rabbit polyclonal anti-HP1021 antibody.

H. pylori COM ΔCys

H. pylori COM ΔCys mutants (N6 COM ΔCys and 26695 COM ΔCys) were constructed as follows (Supplementary Figure S11D). The *cat* cassette was amplified by PCR using the P77-P78 primer pair and pUC18HP1021com plasmid DNA as a template. The P77-P78 primers introduced short sequences to the *cat* cassette homologous to the 5' and 3' flanking regions of the *aphA-3* gene of the pGEM-T Easy HP1021ΔCys plasmid. The amplified DNA fragment was purified using a Gel-Out DNA purification kit (A&A Biotechnology). The *cat* cassette and the pGEM-T Easy HP1021ΔCys plasmid were used to transform *E. coli* BW2513/pkD46 to yield pGEM-T Easy COM HP1021ΔCys via lambda Red recombineering (3). *H. pylori* N6 and 26695 strains were transformed with pGEM-T Easy COM HP1021ΔCys, and transformants were selected by plating on Columbia blood agar plates supplemented with chloramphenicol. The allelic exchange was verified by PCR using P57-P58 primers homologous to chromosomal regions external to the designed recombination sites. The *HP1021* gene was amplified using P79-P80 primers and sequenced to verify the correct recombination and exchange of the codons for cysteine to the codons for alanine. The synthesis of HP1021ΔCys was verified by Western blot analysis using a rabbit polyclonal anti-HP1021 antibody.

Catalase activity

H. pylori liquid cultures at the end of the logarithmic phase of growth were diluted to OD_{600} = 1 in BBL (approx. 6.5 × 10^8 cells/ml). 10 μl of cells' suspension of each strain were simultaneously mixed with 10 μl of 30% H_2O_2; BBL was used as control. The catalase activity was measured by observation of the production of air bubbles.

Disc diffusion assay

Bacteria were cultured in BBL to OD_{600} = ~1 and then diluted OD_{600} = 0.1 in BBL. Each culture was evenly spread by a cotton swab on CBA plates supplemented with 10% FCS and antibiotic cocktail. Sterile, glass fiber 6-mm discs were placed on plates and 5 µl of tested solutions were dropped on discs: 3% (0.88 M) H_2O_2 (POCH, 885193111), 60 mM diamide (Merck, D3648), 0.1 % (6.6 mM) cumene hydroperoxide (Merck, 247502) in DMSO, 0.1 % (3.8 mM) paraquat dichloride (Merck, 36541) and 0.5 mM sodium hypochlorite (Merck, 1056142500; 7-14% active chlorine). Sodium hypochlorite solutions for assays were made fresh; the pH of solutions were adjusted by the addition of HCl to pH 7 and kept in PBS buffer prior to experiments (4). The diameter of the inhibition zone around the discs was determined after 5 days of incubation under microaerobic conditions. The assays were repeated three times using different *H. pylori* cultures. Significance values were calculated using Student's *t*-test. ns, not significant; *, $P < 0.05$; **, $P < 0.01$.

***In silico* analyses**

The secondary structure was predicted by Phyre2 (5) and visualized by EzMol (6). The final HP1021 model was based on seven templates; 99% of residues were modelled at >90% confidence, three residues were modelled by *ab initio.* The highest-confidence model (100%) was obtained using c5m7nA_ fold template (2-295 residues aligned, 98% coverage, 16% identity). The fold template was taken from a PDB file 5M7N representing a structure of *Brucella abortus* NtrX nitrogen assimilation regulatory protein of a two-component system formed by the histidine kinase NtrY and the response regulator NtrX that participates in sensing low oxygen tension and generating an adaptive response (7). To determine the consensus sequence of the HP1021 box a sequence logo (8) was created using sequences of six HP1021 boxes determined by DMS footprinting: TGTTTCA, TGTTACT, TGTTACT at *oriC2*, TGTTTCT at *oriC1*, TGTTACT and GGTAGCG at the promoter region of *hyuA* (all presented as 5'-3' sequences) (1).

Cystein residue position in HP1021

Typical position (conservation [%])	Alternative position (conservation [%])
C27 (100)	0
C51 (99.8)	C52 (0.2)
C56 (99.8)	C57 (0.2)
C216 (99.8)	C217 (0.2)
C238 (94.9)	C239 (0.2)
C270 (99.4)	C269 (0.2)

Figure S1. The conservation of cysteine residue position or alternative positions of the cysteine residues in HP1021 from 486 *H. pylori* strains.

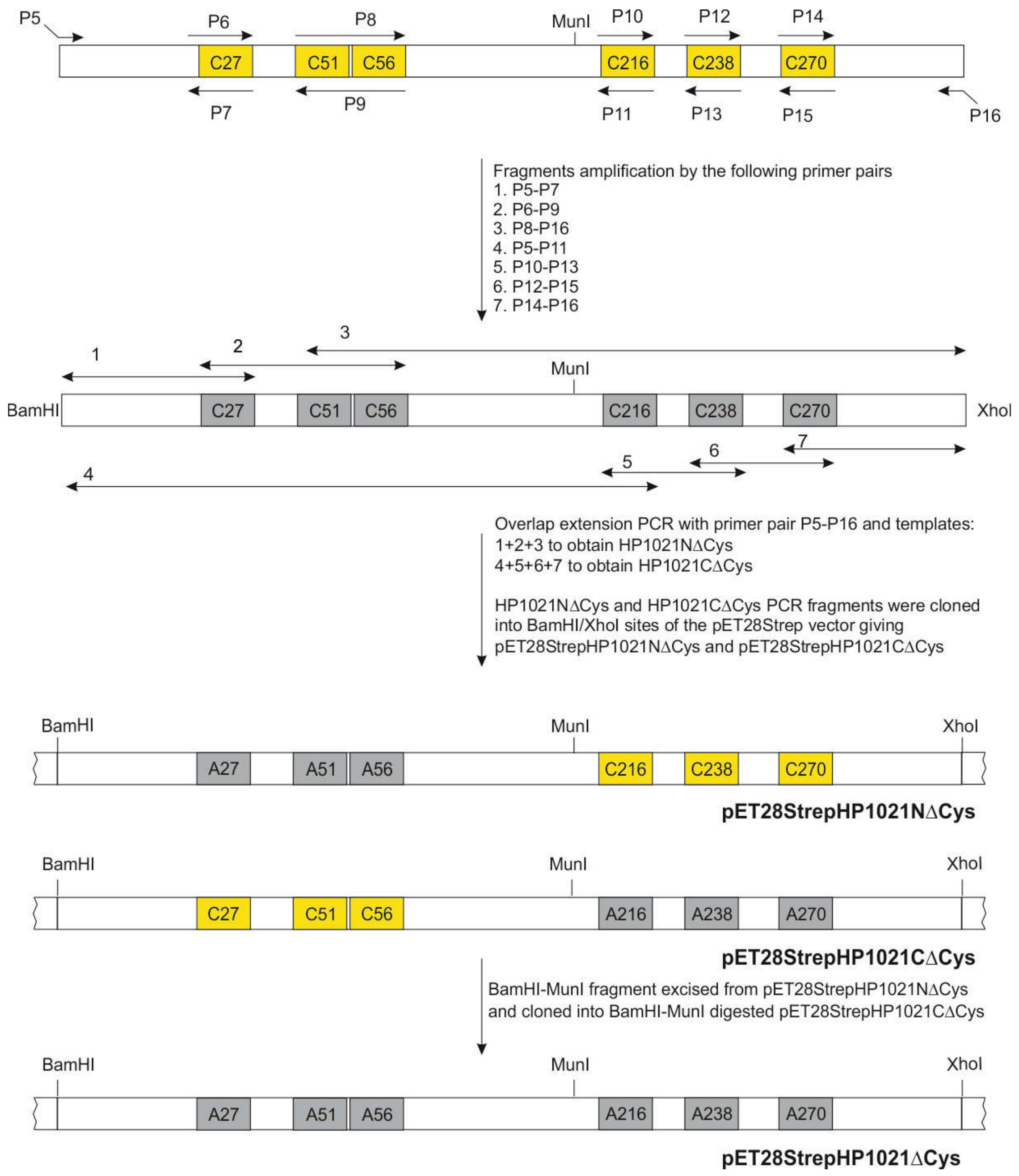

Figure S2. The mutagenesis strategy used to prepare the pET28StrepHP1021ΔCys plasmid to synthesize the cysteine-less recombinant HP1021 variant. Primer sequences are given in Supplementary Table *S2*. The scheme is not to scale.

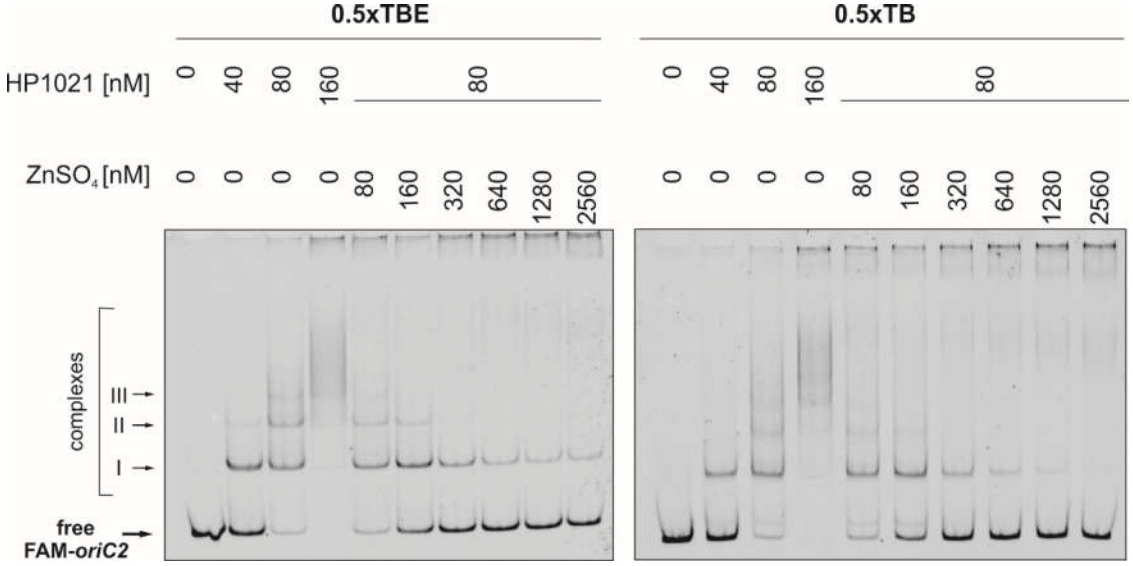

Figure S3. Influence of Zn^{2+} on HP1021 DNA-binding activity. A gel-retardation assay was performed using the FAM-*oriC2* fragment that had been incubated with the indicated amounts of the HP1021 protein variants in the presence or absence of Zn^{2+}. The complexes were separated by electrophoresis on a 4% polyacrylamide gel in 0.5 × TBE or 0.5 × TB buffers (i.e., with or without EDTA, respectively, Materials and Methods). The HP1021-*oriC2* complexes were more stable in 0.5 × TBE buffer than in 0.5 × TB buffer because Zn^{2+}, which destabilizes the complexes, was partially complexed by EDTA present in the buffer. Digital processing was applied equally across the entire image, including controls.

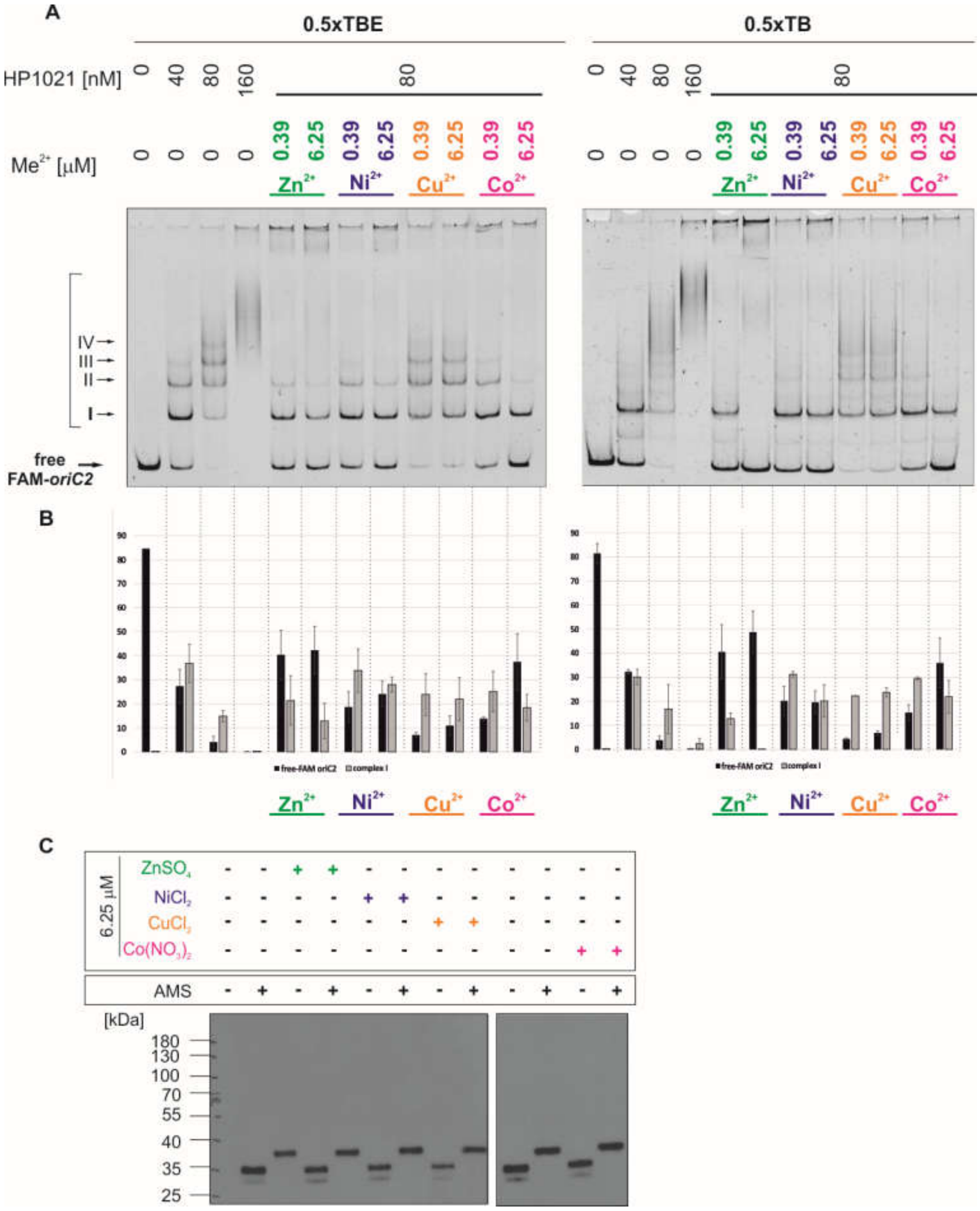

Figure S4. Influence of metal ions on HP1021 DNA-binding activity. (**A**) A gel-retardation assay was performed using the FAM-*oriC2* fragment that had been incubated with the indicated amounts of the HP1021 protein variants in the presence or absence of metal ions. The complexes were separated by electrophoresis on a 4% polyacrylamide gel in 0.5 × TBE or 0.5 × TB buffers (i.e., with or without EDTA, respectively). The HP1021-*oriC2* complexes were more stable in 0.5 × TBE buffer than in 0.5 × TB buffer because Me^{2+}, which destabilize the complexes, were partially complexed by EDTA present in the buffer (**B**) The results of two independent gel-shift analyses were analyzed densitometrically. Free FAM-*oriC2* and complex I were quantified and plotted as a percentage of the signal detected in each lane. Error bars indicate the standard deviation of the two independent analyses. (**C**) Influence of the metal ions on the redox state of the thiol residues in the HP1021 protein. HP0121 was incubated with metal ions under reducing conditions in Tris buffer and labelled with AMS (Materials and Methods). HP1021 was detected in bacterial lysates by a rabbit polyclonal anti-6HisHP1021 IgG. Digital processing was applied equally across the entire images, including controls.

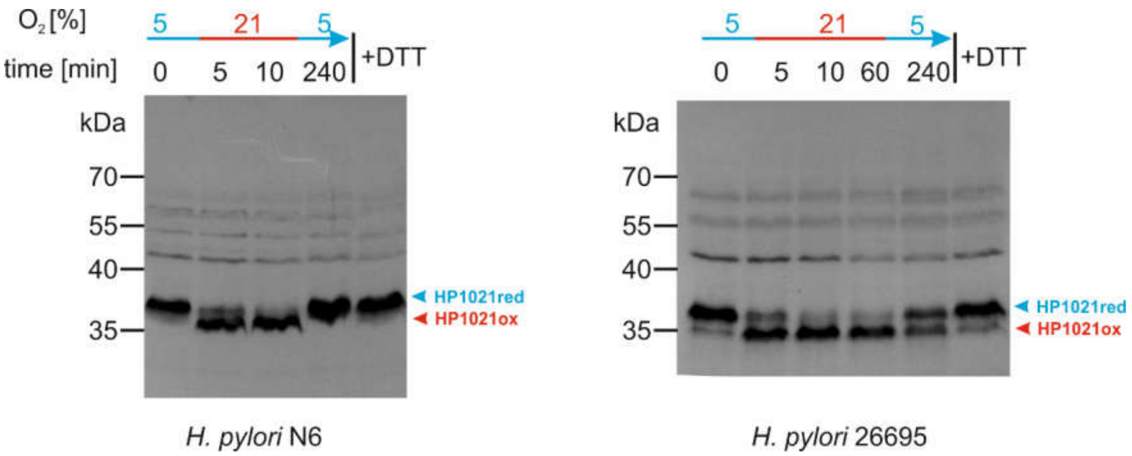

Figure S5. Cysteine residue oxidation in HP1021 in *H. pylori*. Versions of full-length blots presented in Figure 7. The lanes are described similarly as in Figure 7. Digital processing was applied equally across the entire images, including controls.

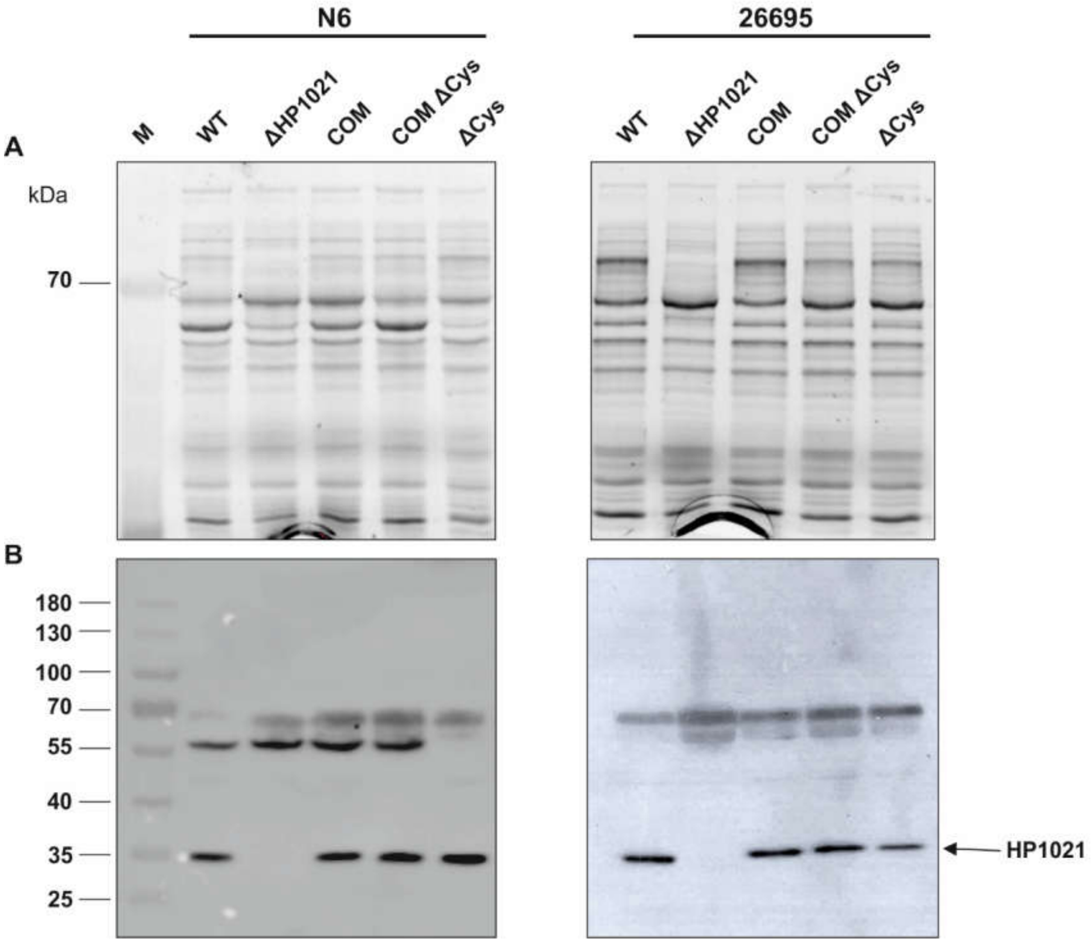

Figure S6. Western blot analysis of HP1021 in *H. pylori* strains. (A) A lysate of each *H. pylori* strain (approximately 1.4 × 10^8 cells per well) was resolved in a 10% SDS-PAGE gel visualized by the TCE-UV method (9). (B) HP1021 was detected in bacterial lysates by a rabbit polyclonal anti-6HisHP1021 IgG. M, PageRuler Prestained Protein Ladder (Thermo Fisher Scientific), this marker is not visualized by the TCE-UV method. Digital processing was applied equally across the entire images, including controls.

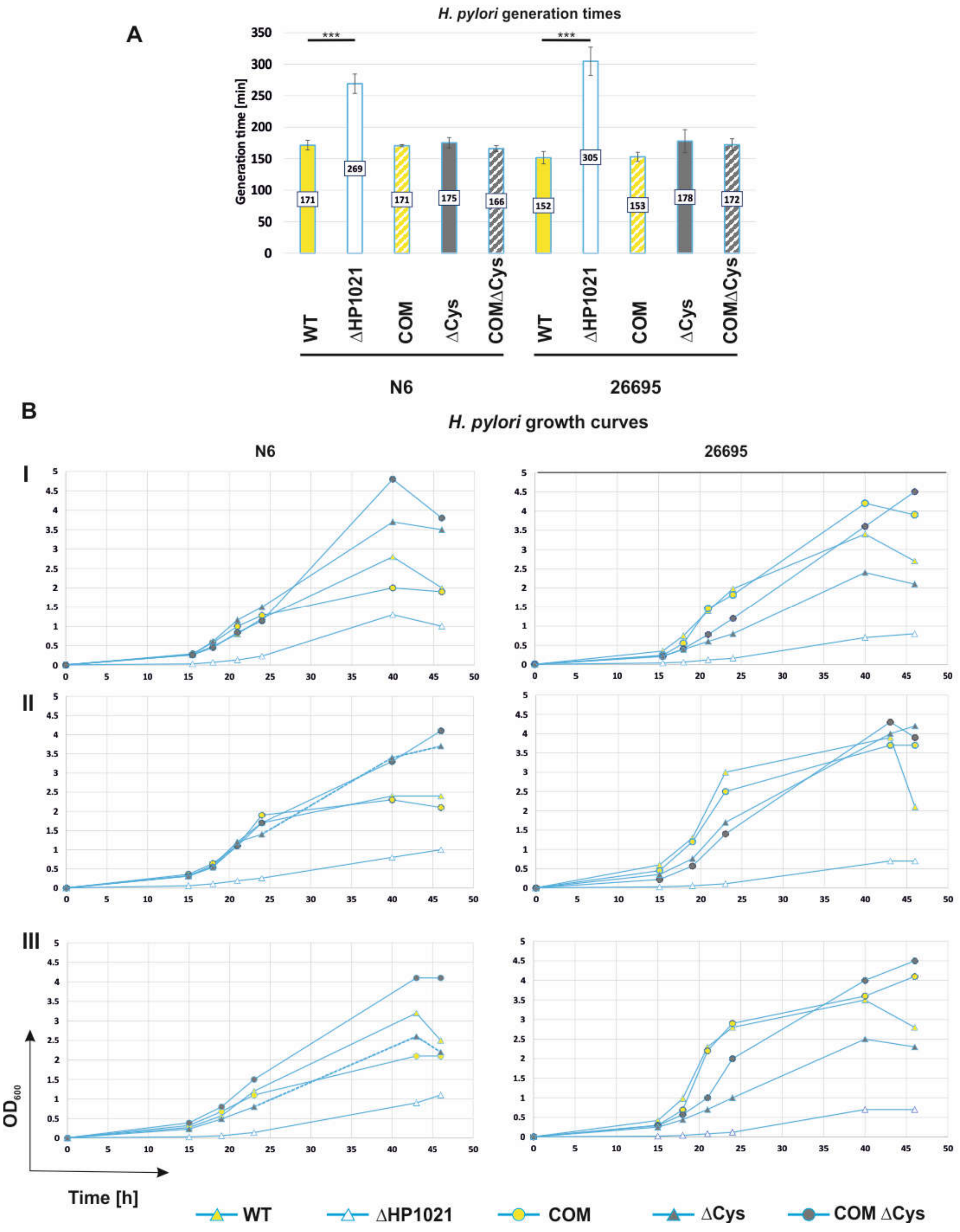

Figure S7. *H. pylori* wild-type and HP1021 mutant strains growth under microaerobic conditions. (**A**) Generation times were calculated for the *H. pylori* N6 and 26695 wild-type and mutant strains grown in three independent cultures for a time period of approximately 0-20 hours of growth. ***, $P < 0.001$. (**B**). Analysis of *H. pylori* growth under microaerobic conditions. *H. pylori* was inoculated in Brucella broth with an OD_{600} = 0.005 and cultured microaerobically until a stationary phase of growth was reached. The growth curves of three independent replicates are shown.

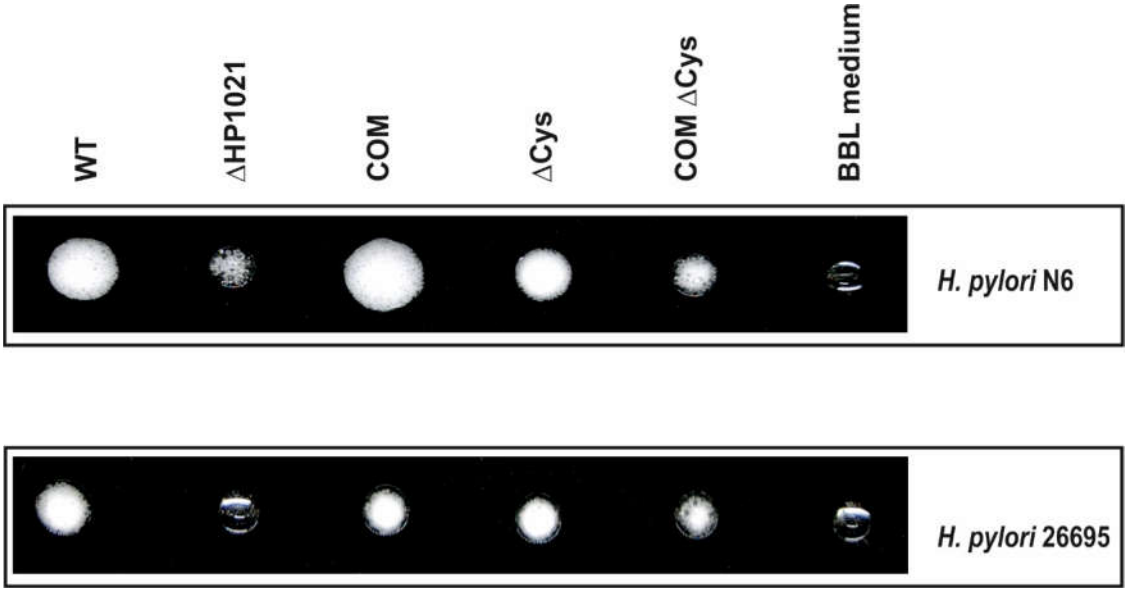

Figure S8. Catalase activity of *H. pylori* wild-type and mutant strains. Liquid growth cultures of the similar cell density (10 μl of *H. pylori* liquid culture of OD_{600} = 1, approx. 6.5 × 10^6 cells) were treated with an equal volume of 30% H_2O_2. Air bubbles produced by catalase are visible as white spots, while translucent liquid drop indicates the lack of catalase activity.

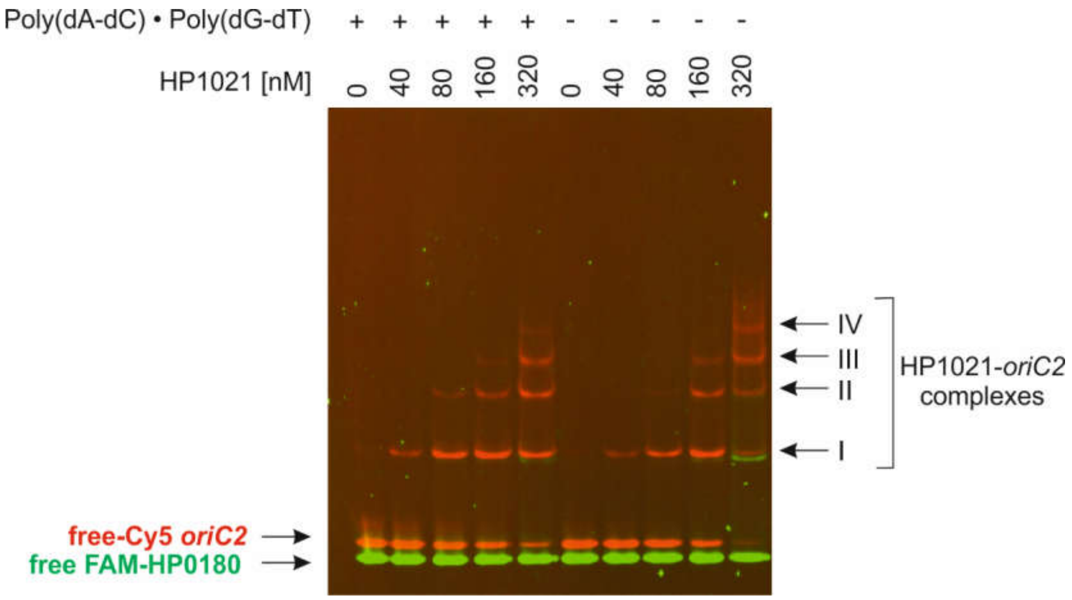

Figure S9. Comparison of the affinity of HP1021 to the *oriC2* subregion and a fragment of HP0180 gene. EMSA was performed using the Cy5-labeled *oriC2* and the FAM-labelled HP0180 gene fragment, which were incubated with HP1021. When indicated, an unspecific competitor DNA was added to the reaction mixture. *OriC2-HP1021* complexes were formed at lower HP1021 concentrations (40 nM) than HP0180-HP1021 complexes (160-320 nM); almost all *oriC2* fragments were bound by HP1021 at 320 nM concentration, while the majority of HP0180 molecules were still unbound at the highest HP1021 concentration used in the analysis. Thus HP0180 DNA fragment is bound with a much lower affinity than *oriC2*. Digital processing was applied equally across the entire image, including controls.

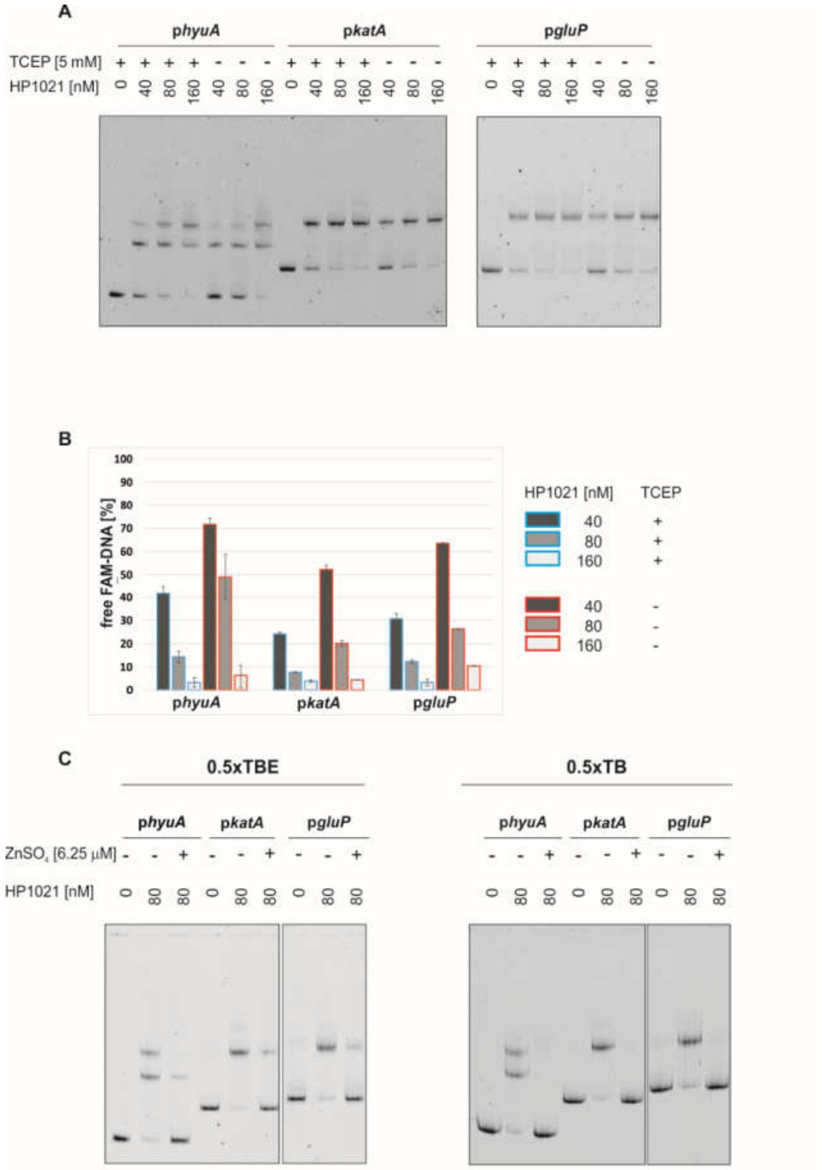

Figure S10. Influence of the redox state and Zn^{2+} on the binding of HP1021 to the putative promoter regions of selected *H. pylori* genes. (**A**) EMSA was performed using the FAM-labeled DNA fragments, which were incubated with the indicated amounts of the HP1021 protein variants in the presence or absence of TCEP. (**B**). The results of two independent gel-shift analyses were analyzed densitometrically. Free FAM-DNA was quantified and plotted as a percentage of the total signal detected in each lane. Error bars indicate the standard deviation of the two independent analyses. (**C**) A gel-retardation assay was performed using the DNA fragments that had been incubated with the indicated amounts of the TCEP-reduced HP1021 protein in the presence or absence of Zn^{2+}. The complexes were separated by electrophoresis on a 4% polyacrylamide gel in 0.5 × TBE or 0.5 × TB buffers (i.e., with or without EDTA, respectively). The HP1021-DNA complexes were more stable in 0.5 × TBE buffer than in 0.5 × TB buffer because Zn^{2+}, which destabilizes the complexes, was partially complexed by EDTA present in the buffer. Digital processing was applied equally across the entire image, including controls.

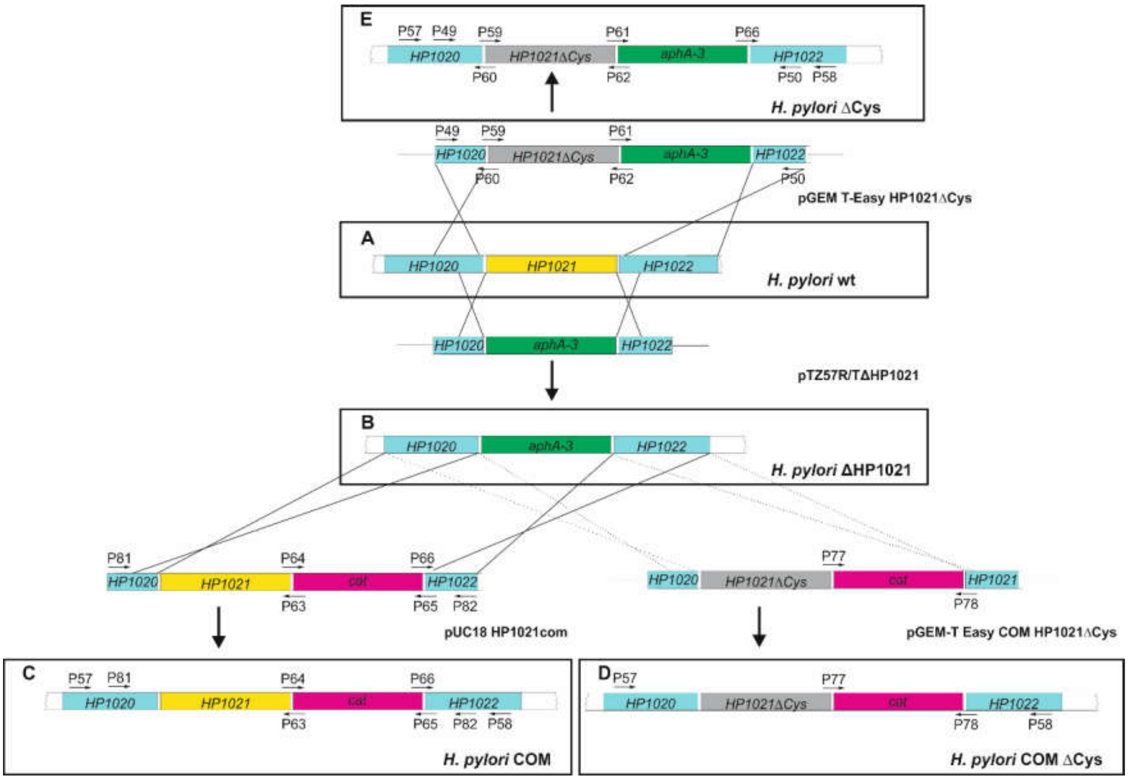

Figure S11. The mutagenesis strategy used to delete or complement HP1021 on the *H. pylori* chromosome. *H. pylori* N6 and 26695 wild-type HP1021 chromosomal loci (**A**) and DNA fragments, which recombined with the *H. pylori* chromosome via double crossing-over to give *H. pylori* ΔHP1021 (**B**) COM (C), COM ΔCys (**D**) and ΔCys (**E**) mutant strains, are shown. *H. pylori* ΔHP1021 was prepared as described earlier (1). Primer sequences are given in Supplementary Table *S2*.

Table S1. Strains, plasmid and proteins used in this study

Strain/plasmid/recombinant proteins	Relevant features	Reference/source
E. coli		
DH5α	*supE*44, *hsdR*17, *recA*1, *endA*1, *gyrA*1, *gyrA*96, *thi-1*, *relA*1	(10)
MC1061	F *araD139* (*ara-leu*)*7696 galE15 galK16*, (*lacX74*) *rpsL hsdR2 mcrA mcrB1*	(11)
BL21	*E. coli* B F-, *ompT*, *hsdS* (rB-, mB-), *gal*, *dcm*.	GE Healthcare
BW2513/pkD46	F-, *Δ(araD-araB)567*, *rph-1*, *ΔlacZ4787*(::rrnB-3), *hsdR514*, *λ*$^{-}$, *Δ(rhaD-rhaB)568,* plasmid pKD46	*E. coli* Genetic Resources at Yale CGSC
H. pylori		
N6	parental strain	(12)
26695	parental strain	(13)
N6 Δ1021	ΔHP1021::aphA-3; N6 with HP1021 exchanged to *aphA*-3 cassette	This work
N6 COM	(ΔHP1021::aphA-3)::(HP1021-cat); N6 ΔHP1021 in which *aphA-3* was exchanged to HP1021 and *cat* cassette	This work
N6 ΔCys	ΔHP1021::HP1021ΔCys-aphA-3; N6 with HP1021 exchanged to mutated HP1021 producing cysteine-less HP1021 protein variant	This work
N6 COM ΔCys	(ΔHP1021::aphA-3)::(HP1021ΔCys-cat); N6ΔHP1021 in which *aphA-3* was exchanged to mutated HP1021 producing cysteine-less HP1021 protein variant and *cat* cassette	This work
26695 Δ1021	ΔHP1021::aphA-3; 26695 with HP1021 exchanged to *aphA*-3 cassette	(1)
26695 COM	(ΔHP1021::aphA-3)::(HP1021-cat); 26695 ΔHP1021 in which *aphA-3* was exchanged to HP1021 and *cat* cassette	This work

26695 ΔCys	ΔHP1021::HP1021ΔCys-aphA-3; 26695 with HP1021 exchanged to mutated HP1021 producing cysteine-less HP1021 protein variant	This work
26695 COM ΔCys	(ΔHP1021::aphA-3)::(HP1021ΔCys-cat); 26695 ΔHP1021 in which *aphA-3* was exchanged to mutated HP1021 producing cysteine-less HP1021 protein variant and *cat* cassette	This work
**Plasmids**		
pGEM-T Easy	TA cloning vector, Amp^R	Promega
pET28a(+)	Bacterial expression vector, Kan^R	Novagen
pUC18	Cloning vector, Amp^R	Thermo Fisher Scientific
pET28Strep	A pET28a(+) derivative, modified by insertion of hybridized P-51 and P-52 oligonucleotides (Supplementary Table S2) between NcoI and BamHI sites. The inserted linker removed the His-tag, thrombin cleavage site and T7 tag sequences and inserted a sequence encoding Streptag (WSHPQFEK, Strep-tag II). The vector was used for expression of recombinant proteins with an N-terminal Strep-tag II (WSHPQFEK) fusion	This work
pET28StrepHP1021	pET28Strep derivative containing the *HP1021* gene amplified with primers P-3/P-4 and cloned between BamHI and XhoI sites	This work
pET28StrepHP1021NΔCys	pET28Strep derivative containing the *HP1021* gene in which codons for cysteine residues C27, C51 and C56 were mutated for alanine codons	This work
pET28StrepHP1021CΔCys	pET28Strep derivative containing the *HP1021* gene in which codons for cysteine residues C216, C238 and C270 were mutated for alanine codons	This work
pET28StrepHP1021ΔCys	pET28Strep derivative containing the *HP1021* gene in which all codons for cysteine residues (C27, C51, C56,	This work

	C216, C238 and C270) were mutated for alanine codons	
pTZ57R/TΔHP1021	pTZ57R/T derivative containing *HP1021* flanking regions for allelic exchange of *HP1021* for *aphA-3*	(1)
pUC18HP1021com	pUC18 derivative containing *HP1021* flanking regions and HP1021 for allelic exchange of *aphA-3* for *HP1021-cat*	This work
pGEM-T Easy HP1021ΔCys	pGEM-T Easy derivative containing *HP1021* gene in which all codons for cysteine residues (C27, C51, C56, C216, C238 and C270) were mutated for alanine codons with flanking regions for allelic exchange of *HP1021* for *HP1021ΔCys-aphA-3*	This work
pGEM-T Easy COM HP1021ΔCys	pGEM-T Easy HP1021ΔCys derivative containing *HP1021* flanking regions and *HP1021*ΔCys-*cat* for allelic exchange of *aphA-3* for *HP1021*ΔCys-*cat*	This work
pori2	A pOC170 derivative containing *oriC*2	(14)
**Recombinant proteins**		
StrepHP1021	recombinant, *H. pylori* HP1021 protein, Strep-tagged at N-terminus, purified from *E. coli*	This work
StrepHP1021ΔCys	recombinant, *H. pylori* HP1021 protein variant in which all 6 cysteine residues (C27, C51, C56, C238, C260 and C270) were mutated for alanine residues	This work

Table S2. Primers used in this study. phos, phosphate; FAM, 6-fluorescein amidite.

Oligo	Sequence (5'->3')
P3	GCGGATCCATGAAAATCTTAATCATTGAAGA
P4	GCCTCGAGTTATTTGCGCGGTAAGTTATATT
P5	GACAGCAAATGGGTCGCGGATCC
P6	GATTTAGGGCATTTTGCTGAGATCATCTCTAGC
P7	GCTAGAGATGATCTCAGCAAAATGCCCTAAATC
P8	GTTTCTTCTAAAGTGGCCACTCAAGGGCGCGCCGAACATTTTGTGCG
P9	CGCACAAAATGTTCGGCGCGCCCTTGAGTGGCCACTTTAGAAGAAAC
P10	GAATTGGCCCGTTCTGCACCTATTGTTTTGTCC
P11	GGACAAAACAATAGGTGCAGAACGGGCCAATTC
P12	ATTGTGAGCATTGAAGCCGGCAATAAACCCC
P13	GGGGTTTATTGCCGGCTTCAATGCTCACA
P14	GCATTTTTCTTCTACAGCGACCGATACAGAATTAG
P15	CTAATTCTGTATCGGTCGCTGTAGAAGAAAAATGC
P16	GTGGTGGTGGTGGTGGTGCTCGAG
P17	FAM-GGAGTAAGAATAGCTTCGAAT
P18	CATCGATAGGATATCCTGGG
P21	GAAAGAGGTAGTGCGGATGC
P22	ACAGGCGTGTTGTTCCCTAC
P25	AAGCACACAATCCCGCTCGC
P26	CGGCTACCCTTTTGGCGTGG
P27	ATAAAACCGGCGATCCTTCT
P28	GCTTGGATACCCACATGCTT
P35	CTAGCGGATTCTCTCAATGTCAA
P36	GGAGTACGGTCGCAAGATTAAA
P49	GCGATTTTAGGAGCGATTAAAGGG
P50	GAATCTTTTAGGGAGTGTTTTATTAG
P51	phos-CATGGCAAGCTGGAGCCACCCGCAGTTCGAAAAAGGCGCCG
P52	phos-GATCCGGCGCCTTTTTCGAACTGCGGGTGGCTCCAGCTTGC
P53	Biotin-CATGTTTGACAGCTTATCATCG
P54	GCATAAGATCTAGGAGTAAGA
P55	Biotin-CTCTATTTTGAAAACCCCTATTTC
P56	CTATATTTTTTCAATGGTTTAGTGC
P57	GCTCACCACGAGCGGCGATTTG
P58	GCTTTAAAATCTTCATCAAATTGG
P59	GTGATAAAAGGAAGAATTGATGAAAATCTTAATCATTGAA
P60	TTCAATGATTAAGATTTTCATCAATTCTTCCTTTTATCAC
P61	ATAACTTACCGCGCAAATAAGGATCCTGACTAACTAGGAGGAA
P62	AATCCTCCTAGTTAGTCAGGATCCTTATTTGCGCGGTAAGTTAT
P63	CCAGTGATTTTTTTCTCCATTTATCCTCCGTAAATTCCGTTATTTGCGCGGCAAATTAT
P64	ATAATTTGCCGCGCAAATAACGGAATTTACGGAGGATAAATGGAGAAAAAAATCACTGG

P65	GCTTAAGGATTTTCTGCTGCAGCATTATTCCCTCCAGGTA
P66	TACCTGGAGGGAATAATGCTGCAGCAGAAAATCCTTAAGC
P67	ATGATATTTATATGATATTTTTGGG
P68	GGAGTAAGAATAGCTTCGAATTTGCGTCTTTCATTTAGACTCC
P69	TGCAATAAAACAGGACCTCTAG
P70	GGAGTAAGAATAGCTTCGAATAATTATTATTAAAACCAGATTAAAA
P73	TTTAACCGTGGTGATGATGC
P74	GGAGTAAGAATAGCTTCGAATGAATAGCGCTGTCAAACTCC
P75	GGAGTAAGAATAGCTTCGAATCTCTATTTTGAAAACCCCTATTT
P77	GGGAAAAACGCCGGAAATATAACTTACCGCGCAAATAA
P78	CCACAAAAAACAGGGCTTAAGGATTTTCTG
P79	GGATTGCGATTTGGGTTAAAGGC
P80	GGAAACTCTCTCTGCCCCTAGTG
P81	GTCGACTCTAGAGGATCCCCATTAAAGGGGGGGATATTGG
P82	TGAATTCGAGCTCGGTACCCGTAAAAGAGTTCTTGAATGCAT
P83	Cy5-CCGCTTTCAATTCAAGTGAATG

Table S3. Analysis of transcription of selected *H. pylori* genes under microaerobic and oxidative stress conditions. RT-qPCR results present a transcription of genes in cells from three independent *H. pylori* cultures (FC I. II and III). FC - fold change; SD - standard deviation.

H. pylori N6 strains			WT		Δ1021		COM-1021		ΔCys-1021		CysCOM-1021	
21% O_2 exposition time [min]			0	20	0	20	0	20	0	20	0	20
Gene	*fecA3*	FC I	1.00	1.54	20.11	22.79	0.93	1.61	4.50	6.45	18.38	25.02
		SD I	0.10	0.09	0.70	0.81	0.04	0.15	0.13	0.29	0.57	0.15
		FC II	1.00	1.93	21.16	15.31	0.60	1.49	2.96	3.66	2.96	6.70
		SD II	0.07	0.19	0.74	1.30	0.05	0.12	0.62	0.37	0.22	0.12
		FC III	1.00	2.57	27.79	34.78	0.95	2.23	5.34	6.65	11.13	20.73
		SD III	0.05	0.42	2.28	2.37	0.06	0.08	0.73	0.28	0.64	0.08
	gluP	FC I	1.00	4.16	3.28	4.44	0.91	2.31	1.76	4.13	19.63	25.11
		SD I	0.09	0.23	0.21	0.31	0.04	0.10	0.10	0.32	0.96	1.62
		FC II	1.00	3.60	9.78	6.18	0.46	2.25	4.04	2.98	2.65	4.95
		SD II	0.06	0.20	0.81	0.17	0.04	0.16	0.43	0.43	0.15	0.34
		FC III	1.00	3.14	4.87	6.42	0.93	2.18	3.34	3.56	6.44	16.72
		SD III	0.04	0.48	0.40	0.50	0.07	0.16	0.23	0.17	0.36	1.61
	katA	FC I	1.00	0.83	0.31	0.39	1.04	0.91	0.82	1.74	0.35	0.43
		SD I	0.11	0.06	0.04	0.03	0.07	0.04	0.03	0.08	0.01	0.01
		FC II	1.00	0.83	0.23	0.39	1.02	0.91	0.83	1.70	0.37	0.42
		SD II	0.21	0.16	0.09	0.08	0.18	0.17	0.18	0.28	0.10	0.07
		FC III	1.00	1.03	0.41	0.48	1.19	0.86	1.18	1.17	0.08	0.22
		SD III	0.06	0.15	0.04	0.03	0.09	0.04	0.11	0.11	0.00	0.02

SI References

1. Donczew,R., Makowski,Ł., Jaworski,P., Bezulska,M., Nowaczyk,M., Zakrzewska-Czerwińska,J. and Zawilak-Pawlik,A. (2015) The atypical response regulator HP1021 controls formation of the Helicobacter pylori replication initiation complex. *Mol. Microbiol.*, **95**, 297–312.
2. Zawilak-Pawlik,A., Zarzecka,U., Żyła-Uklejewicz,D., Lach,J., Strapagiel,D., Tegtmeyer,N., Böhm,M., Backert,S. and Skorko-Glonek,J. (2019) Establishment of serine protease htrA mutants in Helicobacter pylori is associated with secA mutations. *Sci. Rep.*, **9**, 11794.
3. Datsenko,K.A. and Wanner,B.L. (2000) One-step inactivation of chromosomal genes in Escherichia coli K-12 using PCR products. *Proc. Natl. Acad. Sci. U. S. A.*, **97**, 6640–6645.
4. Perkins,A., Tudorica,D.A., Amieva,M.R., James Remington,S. and Guillemin,K. (2019) Helicobacter pylori senses bleach (HOCl) as a chemoattractant using a cytosolic chemoreceptor. *PLoS Biol.*, **17**.
5. Kelley,L.A., Mezulis,S., Yates,C.M., Wass,M.N. and Sternberg,M.J.E. (2015) The Phyre2 web portal for protein modeling, prediction and analysis. *Nat. Protoc.*, **10**, 845–858.
6. Reynolds,C.R., Islam,S.A. and Sternberg,M.J.E. (2018) EzMol: A Web Server Wizard for the Rapid Visualization and Image Production of Protein and Nucleic Acid Structures. *J. Mol. Biol.*, **430**, 2244–2248.
7. Fernández,I., Cornaciu,I., Carrica,M. del C., Uchikawa,E., Hoffmann,G., Sieira,R., Márquez,J.A. and Goldbaum,F.A. (2017) Three-Dimensional Structure of Full-Length NtrX, an Unusual Member of the NtrC Family of Response Regulators. *J. Mol. Biol.*, **429**, 1192–1212.
8. Crooks,G.E., Hon,G., Chandonia,J.-M. and Brenner,S.E. (2004) WebLogo: A Sequence Logo Generator. *Genome Res.*, **14**, 1188–1190.
9. Ladner,C.L., Yang,J., Turner,R.J. and Edwards,R.A. (2004) Visible fluorescent detection of proteins in polyacrylamide gels without staining. *Anal. Biochem.*, **326**, 13–20.
10. Sambrook,J. and Russell,D.W. (2001) Molecular Cloning: A Laboratory Manual Cold Spring Harbor Laboratory Press.
11. Casadaban,M.J. and Cohen,S.N. (1980) Analysis of gene control signals by DNA fusion and cloning in Escherichia coli. *J. Mol. Biol.*, **138**, 179–207.
12. Ferrero,R.L., Cussac,V., Courcoux,P. and Labigne,A. (1992) Construction of isogenic urease-negative mutants of Helicobacter pylori by allelic exchange. *J. Bacteriol.*, **174**, 4212–4217.
13. Tomb,J.F., White,O., Kerlavage,A.R., Clayton,R.A., Sutton,G.G., Fleischmann,R.D., Ketchum,K.A., Klenk,H.P., Gill,S., Dougherty,B.A., *et al.* (1997) The complete genome sequence of the gastric pathogen Helicobacter pylori. *Nature*, **388**, 539–547.
14. Donczew,R., Weigel,C., Lurz,R., Zakrzewska-Czerwinska,J. and Zawilak-Pawlik,A. (2012) Helicobacter pylori oriC--the first bipartite origin of chromosome replication in Gram-negative bacteria. *Nucleic Acids Res.*, **40**, 9647–9660.
